# *Coxiella burnetii* Transcriptional Analysis Reveals Serendipity Clusters of Regulation in Intracellular Bacteria

**DOI:** 10.1371/journal.pone.0015321

**Published:** 2010-12-21

**Authors:** Quentin Leroy, Kevin Lebrigand, Fabrice Armougom, Pascal Barbry, Richard Thiéry, Didier Raoult

**Affiliations:** 1 Unité de Recherche en Maladies Infectieuses et Tropicales Emergentes, CNRS-IRD, UMR 6236, Faculté de Médecine, Université de la Méditerranée, Marseille, France; 2 Institut de Pharmacologie Moléculaire et Cellulaire (IPMC), UMR 6079 CNRS/UNSA, Sophia Antipolis, France; 3 Unité de Pathologie des Ruminants, Agence Française de Sécurité Sanitaire des Aliments (AFSSA) Sophia Antipolis, France; Charité-University Medicine Berlin, Germany

## Abstract

*Coxiella burnetii*, the causative agent of the zoonotic disease Q fever, is mainly transmitted to humans through an aerosol route. A spore-like form allows *C. burnetii* to resist different environmental conditions. Because of this, analysis of the survival strategies used by this bacterium to adapt to new environmental conditions is critical for our understanding of *C. burnetii* pathogenicity. Here, we report the early transcriptional response of *C. burnetii* under temperature stresses. Our data show that *C. burnetii* exhibited minor changes in gene regulation under short exposure to heat or cold shock. While small differences were observed, *C. burnetii* seemed to respond similarly to cold and heat shock. The expression profiles obtained using microarrays produced in-house were confirmed by quantitative RT-PCR. Under temperature stresses, 190 genes were differentially expressed in at least one condition, with a fold change of up to 4. Globally, the differentially expressed genes in *C. burnetii* were associated with bacterial division, (p)ppGpp synthesis, wall and membrane biogenesis and, especially, lipopolysaccharide and peptidoglycan synthesis. These findings could be associated with growth arrest and witnessed transformation of the bacteria to a spore-like form. Unexpectedly, clusters of neighboring genes were differentially expressed. These clusters do not belong to operons or genetic networks; they have no evident associated functions and are not under the control of the same promoters. We also found undescribed but comparable clusters of regulation in previously reported transcriptomic analyses of intracellular bacteria, including *Rickettsia* sp. and *Listeria monocytogenes*. The transcriptomic patterns of *C. burnetii* observed under temperature stresses permits the recognition of unpredicted clusters of regulation for which the trigger mechanism remains unidentified but which may be the result of a new mechanism of epigenetic regulation.

## Introduction

*C. burnetii* is a Gram-negative intracellular γ-proteobacterium that causes Q fever, a zoonotic disease with a worldwide distribution [1]. Q fever can manifest as an acute or chronic illness. Acute Q fever is typically a self-limiting febrile illness during which pneumonia or hepatitis can occur, whereas chronic Q fever is a severe illness that may cause patients to present endocarditis, vascular infection, osteomyelitis and chronic hepatitis [1]. The major route of contamination with *C. burnetii* is as an aerosol. *C. burnetii* displays antigenic variation in its lipopolysaccharides [2]. In phase I, the bacterium is highly infectious, and this corresponds to the natural phase found in animals, humans and arthropods, whereas phase II, which is not very infectious, presents truncated lipolysaccharides and can be obtained after several passages in cell culture or embryonated eggs [1]. The *C. burnetii* genome was sequenced in 2003, and its size is approximately 2 Mbp with a plasmid of approximately 38 kbp [3]. Recently, five new isolates of *C. burnetii* were sequenced [4].

*C. burnetii* displays a complex intracellular cycle, leading to the formation of spore-like forms [5]. McCaul and Williams have proposed the terms “small-cell variant” (SCV) and “large-cell variant” (LCV) to differentiate the two *C. burnetii* cell forms observed in persistently infected cells [6]. SCV are metabolically inactive and resistant to osmotic pressure and correspond to the extracellular form of the bacterium. SCV attach to the eukaryotic cell membrane to enter phagocytic cells. After phagolysosomal fusion, acid activation of the metabolism of SCV may lead to the formation of LCVs. Both LCV and SCV have a typical bacterial Gram-negative cell wall with two layers separated by the periplasmic space. However, a dense material fills the periplasmic space in SCV. This material is composed of proteins and peptidoglycans and may explain the increased resistance of SCV to environmental conditions [7]. The extracellular forms of *C. burnetii* resist environmental conditions such as desiccation and low or high pH, chemical products such as ammonium chloride, disinfectants such as 0.5% sodium hypochlorite, and UV radiation [1], [8].

Temperature change is the most common stress that all living organisms encounter in natural habitats. To overcome critical situations that could be generated by extreme temperatures, bacteria have evolved complex and specific mechanisms that are referred to as cold shock and heat shock responses [9]. Intracellular bacteria exhibit small genomes that show an evolutionary tendency toward genomic reduction, which could be associated with a lower adaptation capacity to environmental changes [10]–[12]. A number of intracellular bacteria have been observed to adapt to environmental changes, including *T. whipplei* and *Rickettsia* sp. [13]–[16]. Different obligate intracellular bacteria have exhibited the expression of specific genes encoding chaperone proteins and proteases that regulate the misfolding of proteins under stress conditions and alarmone accumulation. A previous transcriptional microarray study has been performed to improve an axenic medium for the *C.burnetii* culture [17].

Coordinated virulence gene expression is critical for bacteria during the course of infection. Global transcriptomic approaches have highlighted epigenetic mechanisms associated with bacterial pathogenicity. Cossart et al. showed that noncoding RNA (ncRNA) called small RNA (sRNA) was associated with *Listeria monocytogenes* pathogenicity through use of tilling microarray technology [18]. More recently, a sRNA microarray approach allowed researchers to discover that 6S RNA is implicated in intracellular multiplication [19]. A bacterial RNA seq study found that *Chlamydia trachomatis* exhibits regulation of ncRNA, including 5′ or 3′ untranslated regions and sRNA, during its cellular cycle [20]. These ncRNAs are involved in mechanisms that target gene regulation [21]–[24]. These levels of regulation show that bacterial gene regulation seems to be much more complicated than suggested by the promoter-and-transcription-factor paradigm.

Here, the early adaptive responses and the regulation mechanisms of *C. burnetii* exposed to various sudden temperature shifts were investigated using a whole-genome microarray. We also focus on the specific regulation mechanisms of *C. burnetii* and other intracellular bacteria to adapt in response to environmental stress.

## Results

### Microarray experiments

**Figure 1:**
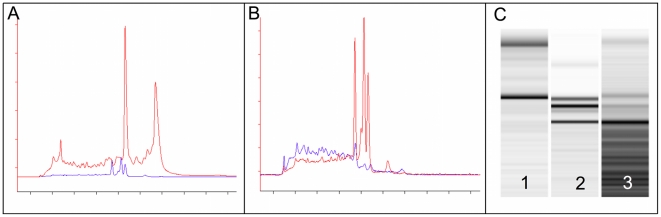
Eukaryotic RNA depletion and the atypical profile of *C. burnetii* rRNA. (A) This figure represents the electrophoregram showing the overlap of total RNA after RNA extraction and bacterial RNA after eukaryotic RNA depletion. (B) This figure represents the electrophoregram showing the overlap of bacterial RNA after eukaryotic RNA depletion RNA and bacterial mRNA after bacterial rRNA depletion. (C) This figure represents the gel-like representation of the fractions obtained after the different RNA depletions.

The complete transcriptional profile of *C. burnetii* exposed to stress temperatures was determined using a whole-genome microarray. Our microarray was spotted in quadruplicate and contained 1990 gene probes that corresponded to ∼98.7% of the coding sequences of this species. Our microarray was validated by self-comparison with genomic DNA and cDNA hybridization (data not shown). In our experimental design, the reference group corresponded to the Nine Mile strain growing at 35°C in normal conditions, while the test group corresponded to the Nine Mile strain exposed to stress temperatures for 30 or 60 min. Bacteria were submitted to stress temperatures of 4 or 42°C, which represent the cold shock (CS) or heat shock (HS), respectively. RNA from bacteria and L929 cells were extracted simultaneously to avoid changes in transcriptomic profile after the bacterial purification process. Eukaryotic RNA was depleted using the MicrobEnrich Kit, which is based on a subtractive hybridization strategy. We found an atypical profile for *C. burnetii* RNA (Figure 1). The cDNA was amplified using random nucleotides and the highly processive phi29 polymerase. The hybridizations were performed in triplicate with three independent cultures. Quantification and *t*-test analyses were applied to determine the genes that were differently expressed at a significant level of confidence of above 95% with a 2-fold cut-off (Table S1). To confirm the global response of the Nine Mile strain, RT-PCR was performed (Table S2).

### General overview

**Figure 2:**
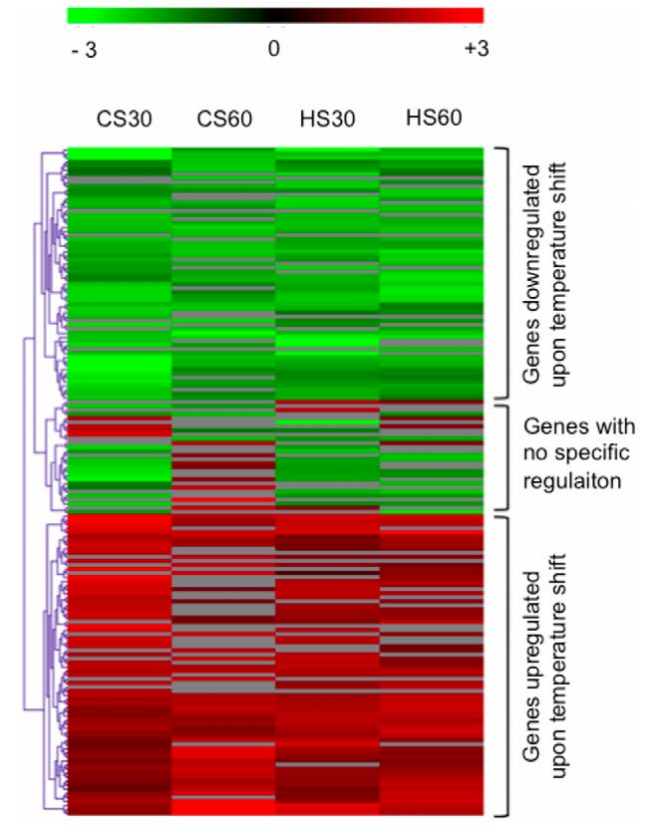
Hierarchical clustering of differentially expressed genes. CS30, CS 60, HS30 and HS60 represent the cold shock stress for 30 min and 60 min and the heat shock stress for 30 min and 60 min, respectively. Green plots represent genes that are downregulated upon temperature stress, red plots represent genes that are upregulated upon temperature stress, and gray plots represent genes with variable regulation observed in biological replicates (p>0,05).

The differentially expressed genes and transcriptomic profile of *C. burnetii* grown at 35°C and then submitted either to heat shock (42°C) or to cold shock (4°C) for 30 min or 1 h, respectively, are shown in Table S1. Our transcriptomic analysis of the *C. burnetii* response to stress temperatures revealed the differential expression of 190 genes, including 140 genes for the CS treatment (85 for 30 min and 62 for 60 min of exposure) and 96 genes for the HS treatment (49 genes for 30 min and 58 for 60 min of exposure) (Table S1 and Figure S1). Surprisingly, a clustering analysis of the differentially expressed genes under the four temperature stress conditions showed that only small differences of expression were detectable between the four treatments (Figure 2).

### Functional analysis

**Figure 3:**
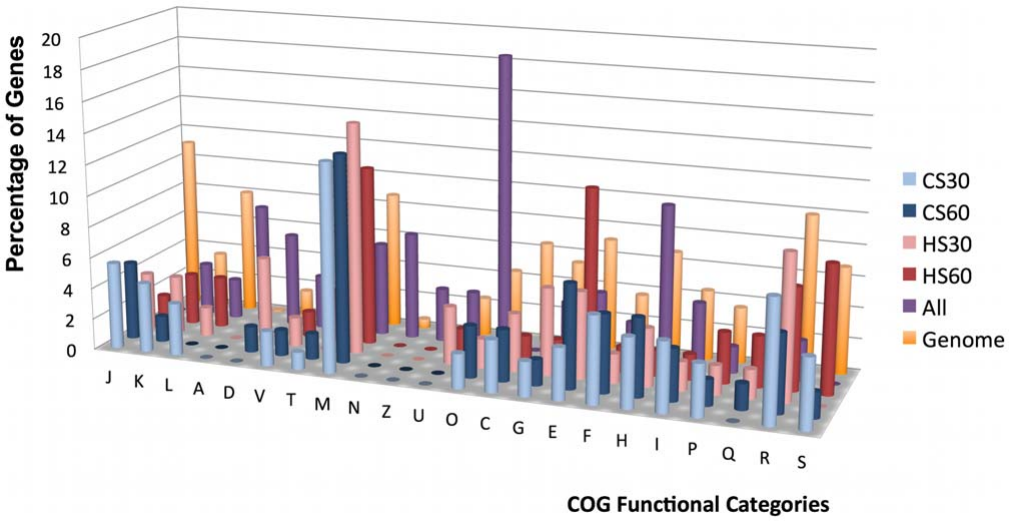
Functional category classification of genes differentially expressed upon exposure to stress temperatures. (A) The figure represents the proportion of genes differentially expressed according to the COG functional classification for each condition and genes differentially expressed at least one time in all of the conditions (All).

We functionally classified the differentially expressed genes according to Cluster of Orthologous Groups database (COG) [25]. We determined the proportion of different functional categories for each condition (Figure 3) and for genes that were differentially expressed in at least one of the stress conditions. The main category of differentially expressed genes was cell wall, membrane and envelope biogenesis (M), with up to 15% following 30 min heat shock. The genes associated with category M encoded outer membrane proteins or proteins involved in the synthesis of lipopolysaccharides, peptidoglycans and mureins. The second principal category observed were genes involved in amino acid transport and metabolism (F), which mostly included genes coding for transport system components (arginine and dipeptides). Nucleotide transport and metabolism (E) genes were also highly regulated, especially under cold shock treatment. In contrast to category E, the intracellular trafficking, secretion, and vesicular transport functional category (U) appeared to be heat shock–specific. Genes involved in cellular functions (transcription (K), replication (L) and translation (J)), representing principal and secondary metabolism, were also differentially expressed. Genes without an associated COG or with an unknown function–associated COG represented approximately 45% of the genes that were differentially expressed.

### Regulon organization

**Figure 4:**
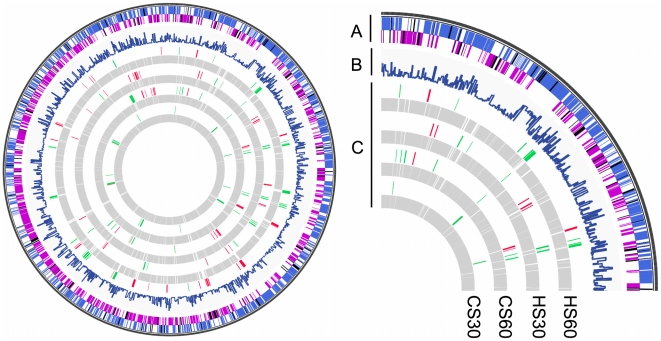
Transcriptional profiles of the early responses to temperature stress. (A) The Outer circle represents the ORFing of *C. burnetii* genome. The blue, purple and black sections represent respectively the spotted ORF from the strand +, the spotted ORF from the strand – and the ORF not spotted. (B) The diagram represents the level of interactions with the other proteins based on String software. (C) The inner circles represent the transcriptomic profiles observed with the four tested conditions. The green, red and gray sections represent respectively the down-regulated, the up-regulated and the not regulated genes.

**Figure 5:**
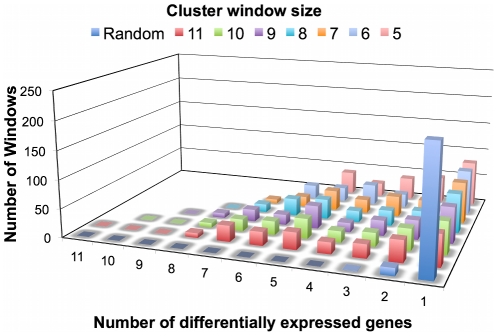
Distribution of differentially expressed genes. This figure represents the total number of differentially expressed genes included in different window sizes (between 5 and 11 genes) that contain differentially expressed genes.

**Figure 6:**
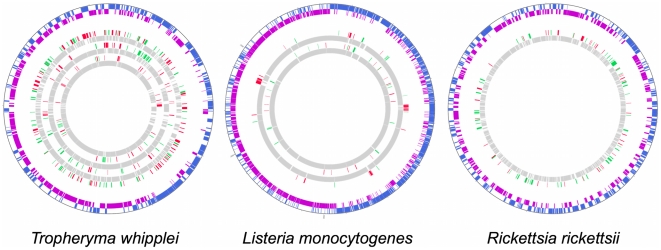
Transcriptional profiles from previously reported analyses of intracellular bacteria. The Outer circle represents the ORFing of the different intracellular bacteria. The blue and purple sections represent respectively the spotted ORF from the strand + and the spotted ORF from the strand –. The inner circles represent the transcriptomic profiles observed in the different studies. The green, red and gray sections represent respectively the down-regulated, the up-regulated and the not regulated genes.

Figure 4 shows that targeted large zones of the bacterial chromosome were simultaneously regulated under stress conditions. We analyzed the structure of these clusters of regulation. Each cluster contained between 5 and 11 genes, at least half of which were differentially expressed (Figure S2). These clusters contained genes that are not necessarily organized into operons, and they can be found on both genomic strands. To check whether this clustering pattern was statistically significant, we split the genome into windows of 5 to 11 genes and counted the number of differentially expressed genes in each. We included in the clusters of regulation all of the windows in which at least the half of genes were found differentially expressed. We found that these clusters of regulation contained differentially expressed genes that were significantly associated compared to a random distribution in the genome (Figure 5 and Table S3). Although the genes mostly occurred in complete operons, single ORFs and incomplete operons were also present in some clusters (Figure S2 and Table S4). To elucidate the mechanism of these regulation clusters, we monitored gene functions within the clusters based on COG classification, but we did not find an enrichment of any specific functional category associated with our clusters compared to rest of the genome (Table S5). Finally, we focused on a functional protein association network using the STRING database 8.2 [26]. Based on the number of connections (score >500) per protein, we determined whether the proteins encoded by the genes included in our clusters were specifically connected compared other *C. burnetii* proteins, but no significant differences were found (Figure S3 and Table S6). Though these clusters of regulation included a number of genes that do not have obvious associated functions, we looked for networks that could link our clusters together and help us to understand this organization of gene expression regulation. Analysis of the protein association network showed that the different clusters seemed to be highly connected for the heat and cold shock conditions, but the connections were mostly spatial connections and not functional (Figure S4). We also looked for structural genomic organization homology between *C. burnetii* and other sequenced γ-proteobacteria that are phylogenetically close to this species according to their 16S rRNA sequences (*Legionella* sp. and *Francisella* sp.). The genes implicated in clusters of regulation in *C. burnetii* presented no clearly identified synteny with those of *Legionella* sp. or *Francisella* sp. (Table S7). Finally, we compared the promoter sequences included in our clusters. We aligned the regions from -1000 bp to the translation start site (TSS). The phylogenic trees obtained from these alignments did not show clustering of promoters associated with either up- or downregulation. We also examined predicted promoters using the Neural Network Promoter Prediction method [27]. We did not find any clearer clustering of promoters associated with gene regulation (Figure S5). We also extracted the region from -10 to the TSS for every transcriptional unit and analyzed the CG% of these sequences (Table S8) to look for a correlation between GC% and transcriptional regulation. We observed no correlation between transcriptional regulation and the GC% of the -10 to translational start site sequences. Furthermore, we examined data from transcriptomic studies on other obligate intracellular bacteria. We collected data from the GEO database and Array Express to look for this kind of spatial regulation in other species, and we found that this type of regulation was also present in other species, including *Rickettsial* species [*14]*–[16], [28**], *Tropheryma whipplei* [13] and *Listeria monocytogenes* [*18*] (Table S9). Figure 6 shows that large regions of the genomes of *R. rickettsii*, *T. whipplei* and *L. monocytogenes* can be highly regulated, comparable to the clusters of regulation found here.

## Discussion

In this study, we examined the early response of gene expression patterns in *C. burnetii* to cold and heat shock using a global transcriptional approach based on microarray technology. Microarray-based transcriptional studies for obligate intracellular bacteria have limitations, such as obtaining RNA of sufficient quality and quantity [29], [30]. Bacterial purification from infected cells involves several steps at 4°C, and bacteria are highly sensitive to cold shock [31]. To prevent the treatments from skewing the results, we extracted eukaryotic and prokaryotic RNA simultaneously, and eukaryotic RNA was then removed by subtractive hybridization. We observed an atypical rRNA profile (Figure 1) with three peaks. This atypical profile is due to an insertion sequence in the 23S rRNA gene, as previously described [32], [33]. Considering the 23S rRNA split, we obtained purified *C. burnetii* RNA of good quality. This strategy of eukaryotic RNA depletion coupled with cDNA amplification was previously successfully devised for the global transcriptomic analysis of intracellular bacteria [34]. Finally, the results obtained by qRT-PCR validate our microarray hybridization experiments, which were carried out with bacterial RNA extracted from three independent experiments.

According to our results, *Coxiella burnetii* appears not to be highly sensitive to temperature shifts corresponding to CS and HS. We found few genes that were differentially expressed (around 3±1% per temperature stress conditions). The genes regulated upon exposure to stress temperatures showed minor changes, with up to a 4-fold change in their expression. Thus, we speculate that host cells provide a stable environment and can partially decrease transcriptional responses from occurring in obligate intracellular bacteria. Surprisingly, a clustering analysis of the differentially expressed genes under the four temperature stress conditions examined (Figure 2) shows that only slight differences of expression were detectable between the four conditions (as was previously shown with for *T. whipplei*) [13]. These similar transcriptomic profiles suggest that *C. burnetii* uses identical strategies to protect itself from CS and HS during its early exposure to these conditions within cells (Figure 2).

Even if bacteria do have the capacity to adapt quickly, our study largely reflects the very early and early responses of *C. burnetii* to temperature shifts. Moreover, we point out these transcriptomic profiles reflect that *C. burnetii* could have been under growth arrest. A slowdown in cellular division in *C. burnetii* could be supported by the downregulation of genes coding for the septum placement (*ftsZ*) [35], [36] [37], the segregation of the plasmid (*parB*) [38] and genes associated with cell division (*ftsY*, *gidAB*) [39], [40]. The downregulation of genes implicated in alarmone degradation (*rpoZ*, *spoT and gmK*) [41], [42] indicates a guanosine pentaphosphate or tetraphosphate ((p)ppGpp) accumulation, which is involved in the stringent response and in bacterial sporulation [42]–[44]. The stringent response is classically followed by growth arrest. While most of genes coding for chaperone proteins are generally underexpressed, Hsp90 could be activated *via* HemE [45]. The cell wall and the membrane of *C. burnetii* seem to be modified and are associated with a spherical shape (*merBCD*) [43], [46]. The bacteria also undergo homeostatic maintenance, in which ABC transport and efflux pumps are implicated (*artM*, *artQ*, *opp* system). The decrease in bacterial division coupled with the putative morphological aspects, the changes in the membrane and cell wall, and the homeostatic maintenance could potentially correlate to a transformation of *C. burnetii* into a metabolically inactive sporulation-like form (SCV) [5]. The SCV form seems to be associated with the stress response of *C. burnetii* and could confer on the bacteria strong resistance to environmental changes, such as CS and HS.

Surprisingly, we observed that significantly differentially expressed genes were mostly spatially clustered following exposure to stress temperatures (Figure 3), and we found that these genes were highly significantly spatially associated compared to a random distribution. Then, we hypothesized that this distribution was associated with a transcriptional regulation mechanism. Different levels of bacterial gene expression regulation have been previously characterized, such as organization related to operons and regulons [47]. However, the clustering found in this study was not obviously associated with operon organization. Some genes were even found on the two different DNA strands. The second level of regulation could be related to functional associations and network connections. A study of the *Rickettsia prowazekii* transcriptional response to cold shock found that that only genes associated with posttranscriptional modification, such as protease and chaperon proteins, were differentially regulated [14]. However, our investigation of functional associations using COG classifications and network connections did not allow us to find any obvious associations. Furthermore, a study of genomic organization showed that our clusters of differentially expressed genes were not highly syntenic with those of other bacteria, in particular with phylogenetically closely related bacteria, including *L. pneumophila* and *F. tularensis*. This could indicate a lack of functional selection pressure. Another transcriptional level of regulation is the regulon. A regulon is a collection of genes or operons under regulation by the same regulatory protein. The observed downregulation of the gene coding for the RNA polymerase omega subunit in all of our experimental conditions directed our research toward the regulon phenomenon. We analyzed the downstream intergenic sequences of our differentially expressed genes to look for similarity in promoter patterns. As we mentioned previously, heat shock appears to be involved (p)ppGpp accumulation within these bacteria. ppGpp is known as a transcriptional regulator [48], [49], and DksA, which binds to the RNA polymerase secondary channel, potentiates the effects of (p)ppGpp on transcription. The direct activation or repression of a gene promoter by (p)ppGpp and DksA is dictated by specific DNA sequence motifs [48], [49]. Repressed genes are typically GC rich between the -10 hexamer box and the TSS, whereas activated genes are typically AT-rich in this position. Our analysis of promoter regions did not uncover any correlation of GC content and regulation within the regulated genes or the genes contained in clusters. The observed clustering of differentially expressed genes could not be attributed to (p)ppGpp or DksA regulation associated with the -10 to TTS region of these genes. The promoter analysis does not highlight a putative role associated with regulons. Thus, it is easy to speculate that the regulation observed in this study could be due to epigenetic regulatory factors, or it could be an artifact from our methods.

To confirm the existence of this clustering of differentially expressed genes around the genome, we collected data from transcriptional microarray analyses of different obligate intracellular bacteria that we listed in a recent review [30]. From these data, we easily observed undescribed but comparable clusters of differentially expressed genes in different conditions for *Rickettsia* sp. [15], [16], [28], [50] and *T. whipplei* [13] (Figure 6 and Table S9). These studies have mostly focused on environmental changes. These findings indicate that regulation that can occur under conditions of stress. However, these studies were performed with low-density arrays and could be an artifact of the hybridization or analysis methods used. DNA probes are generally randomly spotted or synthesized on glass surfaces. In this regard, we can eliminate hybridization artifacts. A recent transcriptomic analysis of *L. monocytogenes* was performed using tilling microarrays [18]. Tilling arrays permit the investigation of whole genomes and should clearly reflect transcriptomic profiles. This transcriptomic analysis using tilling arrays for *L. monocytogenes* highlighted clusters of regulation (Figure 6 and Table S9). It is possible that the limited number of RNA-seq studies of intracellular bacteria could explain why we have not observed these regulatory arrangements in previous RNA-seq studies. Here, we only focused on obligate intracellular bacteria and one facultative bacterium, but the observation of these regulatory clusters in different bacteria allowed us to confirm that these clusters appear to be a real, undescribed regulation phenomenon. Though we think that the definition of the observed regulatory clusters depends on the threshold applied to the data, this may be indicative of hot spots for coregulation, independent of operons or strain positions.

These hot spots of regulation do not correspond to classical transcriptomic regulation within the promoter-and-transcription-factor paradigm. We speculate that an epigenetic regulation mechanism is responsible for the clustering of differentially expressed genes. Furthermore, recent studies based on bacterial RNA-seq methods [20], [51] or tilling microarrays [18] have focused on a new level of gene expression regulation. ncRNA epigenetic regulation, including sRNA and riboswitches in 5′ untranslated regions, have been highlighted and associated with bacterial virulence [18]. We can speculate that our differentially expressed genes could be targets of sRNA, and riboswitches could represent a plausible hypothesis to explain our observations. ABC transporters and efflux pumps are differentially expressed by *C. burnetii*. Riboswitches act as sensors and can activate or inhibit transcription in the presence of a specific molecule [52]. We can also hypothesize that there may be other epigenetic factors involved, such as hot spots of DNA methylation or DNA supercoiling, that could decrease the accessibility of transcription factors or RNA polymerase to promoter sequences [47]. Such phenomena are well known in eukaryotic models, such as in ncRNA silencing, and it is easy to speculate that this could be responsible for the clusters of regulation we have observed.

In conclusion, *C. burnetii* appears to be able to rapidly adapt itself to environmental changes such as cold and heat shock by altering the transcription of adapted genes that could be involved in transformation into a sporulation-like form. In bacteria, genes are organized into operons to facilitate the regulation of genes implicated in the same pathway. Here, we found that many of the genes that are differentially expressed upon exposure to temperature stresses are organized into clusters of regulation. Although we have not deciphered the mechanisms underlying these regulation clusters, this phenomenon seems to be widespread in obligate intracellular bacteria. Clustering related to the regulation of gene expression involved in bacterial adaptation could be advantageous for these bacteria. Thus, we will undertake new experiments related to transcriptional responses with longer exposure to stress conditions using technology that is adapted to highlight ncRNA or epigenetic factors (which we could not monitor with the microarray used here) to elucidate the phenomenon of gene regulation by clusters.

## Materials and Methods

### Strain, medium and growth conditions

All experiments were performed with mid-log cultures of *C. burnetii* grown at 35°C on L929 cells in MEM (GIBCO, Invitrogen, Cergy-Pontoise, France) supplemented with 4% SVF (GIBCO) and 1% L-glutamine (GIBCO). For temperature stress experiments, flasks containing infected L929 cells were incubated at 4°C or 42°C for 30 min or 1 h with the Nine Mile I Strain. Infected cells were harvested using glass beads and centrifuged at 7,500 rpm for 10 min. Pellets were frozen using liquid nitrogen and stored at −80°C.

### RNA extraction and purification

Pellets were resuspended in 100 µl of TE supplemented with 10 mg/ml of lysozyme (Euromedex, Souffelweyersheim, France) and incubated for 10 min at room temperature. Total RNA was extracted and purified from resuspended pellets using the RNeasy Mini Kit (Qiagen, Courtaboeuf, France) as recommended by the manufacturer. DNase treatment was performed using the DNA Turbo Free Kit (Ambion, Applied Biosystems, Courtaboeuf, France). Total RNA integrity was checked using the 2100 BioAnalyzer (Agilent Technologies, Palo Alto, CA), and the concentrations were quantified using the NanoDrop (Thermo, Wilmington, USA). Eukaryotic RNA and bacterial rRNA were depleted using the MicrobEnrich kit (Ambion) as previously described [53] and the MicrobExpress kit (Ambion), respectively. The integrity of bacterial RNA was checked using the 2100 BioAnalyzer, and the concentrations were quantified using the NanoDrop.

### RNA labeling for microarray experiments

RNA was reverse-transcribed using M-MLV (Invitrogen, Cergy-Pontoise, France) and random hexamer primers (Invitrogen) as previously described. cDNAs were amplified using the processive polymerase phi29 with the GenomPhi illustrator V2 kit (GE HealthCare Lifescience, Orsay, France). This strategy was previously described [29]. The amplified cDNAs were labeled with the Bioprime Labeling System (Invitrogen) using d-CTP Cy3/5 fluorochromes (GE HealthCare Lifescience). Labeled cDNAs were purified using QIAquick mini kit columns (Qiagen), and the level of incorporation was quantified using the NanoDrop.

### *Coxiella burnetii* whole-genome microarray construction

OligoArray 2.0 [54], [55] was used to design probes based on 2016 CDS extracted from the NC_002971.gb GenBank sequence file. OligoArray 2.0 integrates BLAST analysis against a nonredundant set of sequences and probe secondary structure analyses [56]. Oligonucleotide calculation parameters were set as follows: oligo length from 50- to 52-mers; GC percentage from 35 to 55%; melting temperature from 82 to 86°C. OligoArray 2.0 selected probes with the lowest cross-hybridization and an absence of secondary structure and balanced the set of probes in terms of melting temperature. Oligonucleotides containing five consecutive A, C, G or T were discarded. Following probes design, 1990 probes corresponding to 1990 distinct CDS where selected for synthesis. A total of 100 µmol of each probe were ordered from Sigma–Proligo (Paris, FRANCE) as 5′ amino–modified oligonucleotides. Oligonucleotide stocks were aliquoted for use in microarray fabrication. Oligonucleotides were diluted to a final concentration of 35–50 µM in 35% dimethyl sulfoxide, 100 mM potassium phosphate (pH 8.0). *C. burnetii* 2k microarrays (GEO reference GPL6675) were printed with a ChipWriterProarrayer (Bio-Rad, 1000 Alfred Nobel Drive Hercules, CA) on commercial HydroGel slides (Schott, Hattenbergstr 10 55122 Mainz, Germany) and processed according to the manufacturer's instructions.

### Microarray hybridizations

Hybridization was carried out using two samples of labeled cDNA (75 pmol of each) that were labeled with Cy3 or Cy5 d-CTP. The pooled samples were hybridized using the GE Hybridization Kit (Agilent Technologies) as recommended by the manufacturer. The mixture was applied to a Surhyb 1 array (Agilent Technologies) and hybridized onto the *Coxiella burnetii* array using an Agilent hybridization chamber (Agilent Technologies). Microarrays were hybridized for 17 h at 62°C in a rotating oven. Microarrays were washed using the GE washing buffers (Agilent Technologies) for 5 min with wash buffer 1 at room temperature followed by 1 min with wash buffer 2 at 37°C. Microarrays were dried using a bath of acetonitrile (VWR, Fontenay sous Bois, France). The microarrays were scanned using the microarray scanner C (Agilent Technologies) using XDR at 5 µm resolution.

### Analysis of microarray data

All microarray results have been deposited in the GEO database (http://www.ncbi.nlm.nih.gov/geo/) under GEO series accession number GSE21778. The signal intensity and local background were measured for each spot by analyzing the array pictures with Feature Extractor software (Agilent Technologies). The data filtering and normalization were performed using Midas from the TM4 suite (TIGR). Data normalizations were performed using global normalization and Lowess normalization methods. Normalized data were processed using Tmev software from the TM4 suite (TIGR) with a *t*-test with a p-value of <0.05 and a cut-off of 2 for the fold change [50]. All experiments were conducted three times, which yielded 12 measurements per gene (representing four technical replicates in three biological replicates). The gene expression level was determined by determining the mean of the 12 values obtained for each probe.

### Cluster of regulation analysis

Differentially expressed gene distributions were calculated using windows of 5 to 11 genes. The number of differentially expressed genes was counted in each window, and the distribution of differentially expressed genes was compared to a random distribution. For the synteny analyses, we compared *C. burnetii* to *Legionella* sp. and *Francisella* sp. using the Geneplot application. Geneplot is available on the NCBI website (http://www.ncbi.nlm.nih.gov). Functional analyses were performed using the Cluster of Orthologous Gene classification (COG) [25]. We used the operon organization algorithm available in MicrobesOnline to define transcriptional units [57]. Protein network data were extracted from the program STRING version 8.2 [26]. We used interactions with a score >0.5. We extracted all downstream intergenic sequences (−1000 to 0 bp) for all of the genes and considered as putative promoter sequences all intergenic sequences with a length >50 bp. Promoter prediction has also been performed on putative promoter sequences using the Neural Network Promoter Prediction method [27]. Promoter predictions and downstream intergenic sequences corresponding to our differentially regulated genes were aligned using the Muscle 3.7 program [58]. Phylogenetic trees were built using MEGA 4 software [59]. We extracted all of the downstream intergenic sequences (−10 to 0 bp) for all of the transcriptional units and considered sequences with intergenic sequence lengths >50 bp. We analyzed the CG% of the extracted sequences in comparison to the GC% of intergenic sequences. Statistical analyses were performed using GraphPad Prism version 5 software. All of the collected information on *C. burnetii* genes are listed in Table S4.

### Real-Time RT-PCR

RNA was reverse-transcribed using M-MLV (Invitrogen) and random hexamer primers (Invitrogen) as recommended by the manufacturer. qPCR was performed on cDNAs for targeted transcripts using the Quantitec Probes Kit (Qiagen) with the 7900 HT PCR system (Applied Biosystems). The primers and probes used to perform qPCR were designed based on the five *C. burnetii* sequenced genomes available on the NCBI database. The sequences of primers and probes used are listed in the Table S2. The relative expression ratios of target genes were determined by comparing housekeeping genes (*com1*, *16S*, *rpoB*) with differentially transcribed genes using the software of the 7900 HT qPCR system.

## Supporting Information

Figure S1 Venn diagram of differentially expressed genes.
(PPT)

Figure S2 Clusters of regulation.
(PPT)

Figure S3 *Coxiella burnetii* gene network connections.
(PPT)

Figure S4 Differentially expressed gene networks.
(PPT)

Figure S5 Promoter sequence analysis.
(PPT)

Table S1 List of differentially expressed genes.
(XLS)

Table S2 Table of primers and probes used for qRT-PCR and results.
(XLS)

Table S3 Statistical analysis of the distribution of differentially expressed genes.
(XLS)

Table S4 Genes information**.**
(XLS)

Table S5 Statistical analysis of functional categories within clusters.
(XLS)

Table S6 Statistical analysis of network connections within clusters.
(XLS)

Table S7 Synteny between *C. burnetii* and *Legionella* sp. and *Francisella* sp.
(XLS)

Table S8 Promoter -10 to start site analysis.
(XLS)

Table S9 Clusters of regulation within other obligate intracellular bacteria.
(XLS)
