# Supplementary material for: Coxiella burnetii Transcriptional Analysis Reveals Serendipity Clusters of Regulation in Intracellular Bacteria
Source: PLoS One. 2010 Dec 21;5(12):e15321. doi: 10.1371/journal.pone.0015321 (PMC3006202; doi:10.1371/journal.pone.0015321)
Supplement: Figure S1 — Venn diagram of differentially expressed genes. (PPT) [file pone.0015321.s001.ppt]

## Slide 1
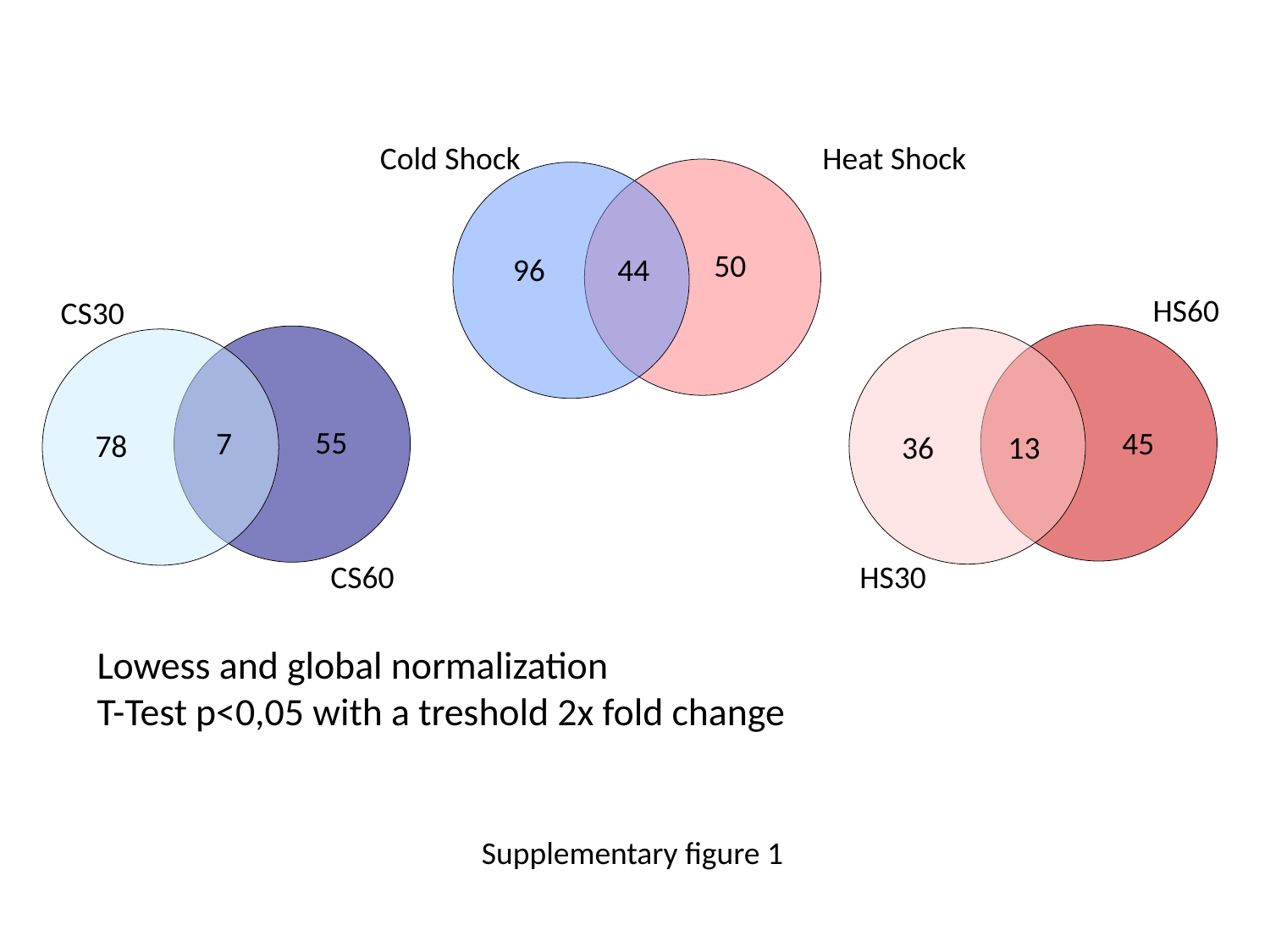

96
44
Cold Shock
Heat Shock
50
HS60
CS30
55
7
45
78
36
13
CS60
HS30
Lowess and global normalization
T-Test p<0,05 with a treshold 2x fold change
Supplementary figure 1
