# Supplementary material for: Coxiella burnetii Transcriptional Analysis Reveals Serendipity Clusters of Regulation in Intracellular Bacteria
Source: PLoS One. 2010 Dec 21;5(12):e15321. doi: 10.1371/journal.pone.0015321 (PMC3006202; doi:10.1371/journal.pone.0015321)
Supplement: Figure S2 — Clusters of regulation. (PPT) [file pone.0015321.s002.ppt]

## Slide 1
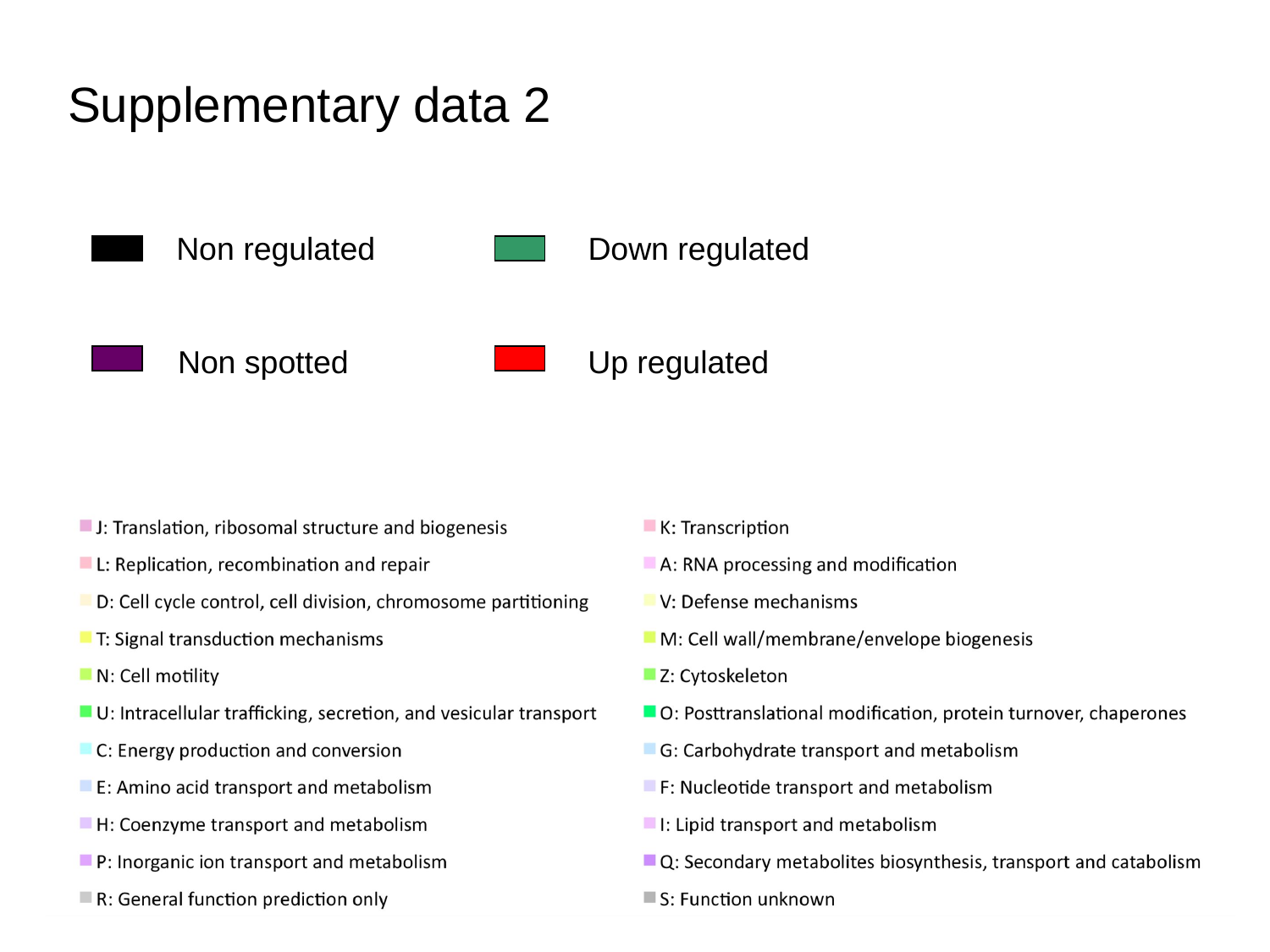

Supplementary data 2
Non regulated
Down regulated
Non spotted
Up regulated

## Slide 2
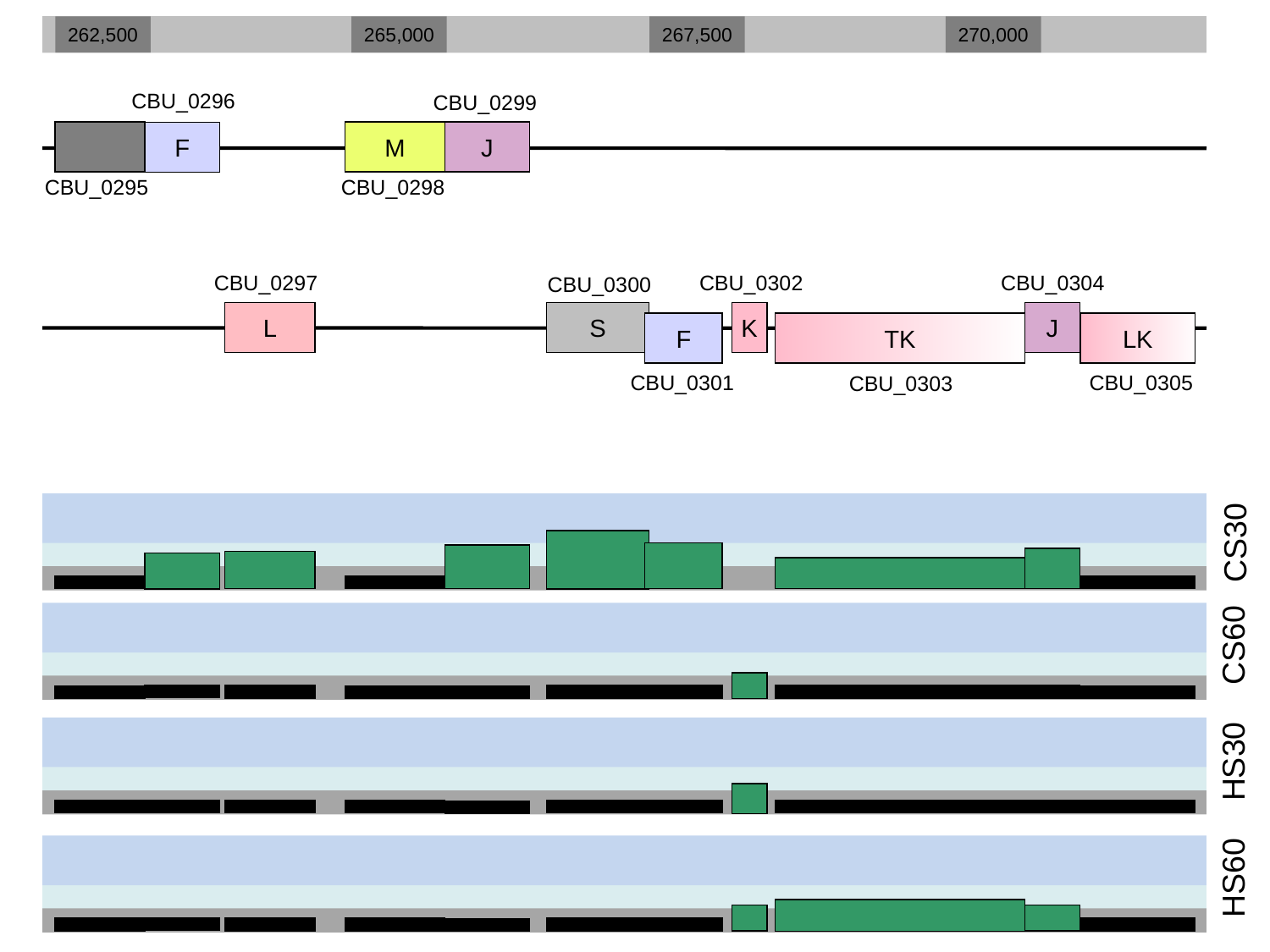

262,500
265,000
267,500
270,000
CBU_0296
CBU_0299
M
J
F
CBU_0295
CBU_0298
CBU_0297
CBU_0302
CBU_0304
CBU_0300
L
S
K
J
F
TK
LK
CBU_0301
CBU_0305
CBU_0303
CS30
CS60
HS30
HS60

## Slide 3
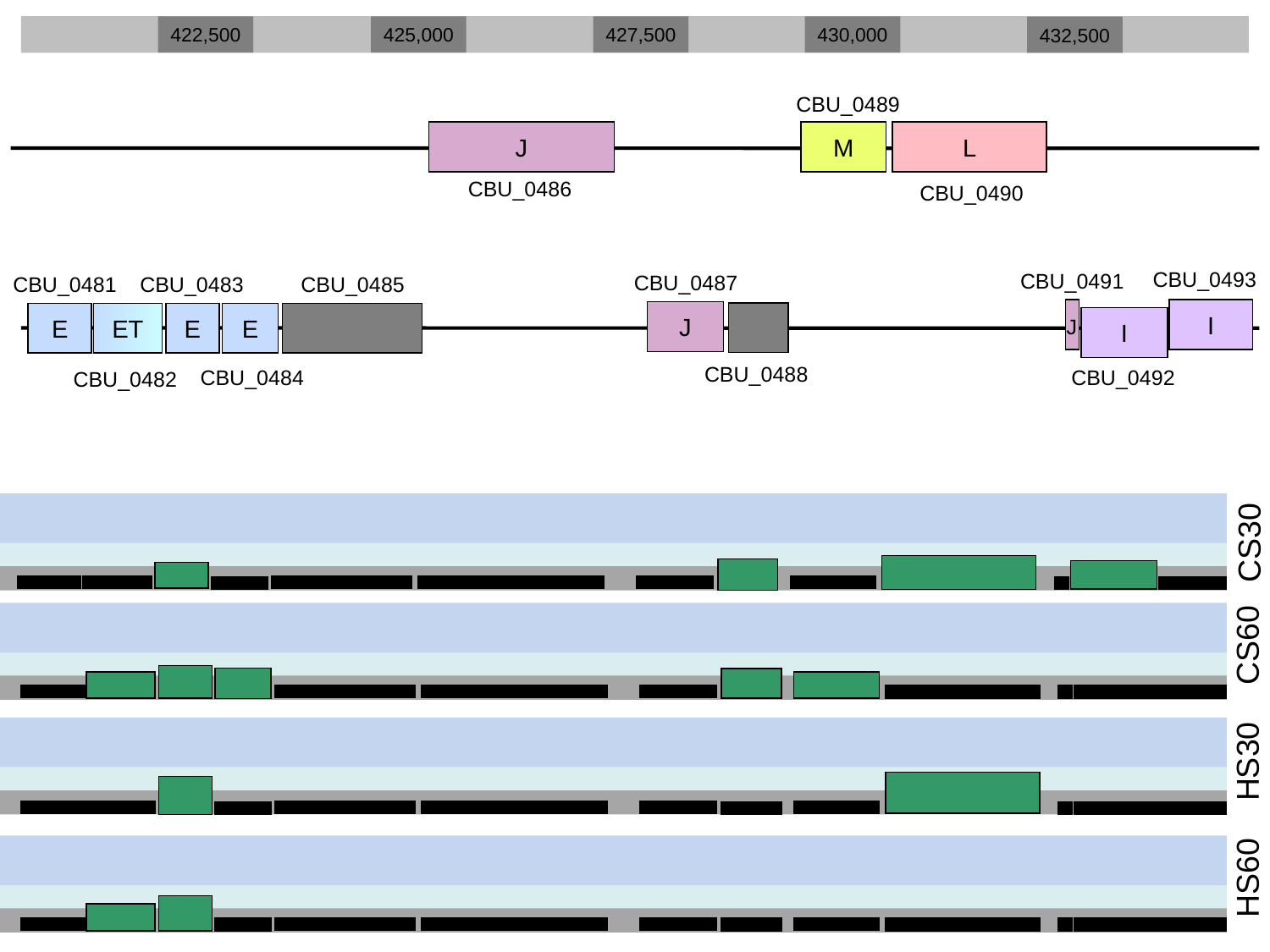

422,500
425,000
427,500
430,000
432,500
CBU_0489
J
M
L
CBU_0486
CBU_0490
CBU_0493
CBU_0491
CBU_0487
CBU_0481
CBU_0483
CBU_0485
I
J
E
ET
E
E
J
I
CBU_0488
CBU_0484
CBU_0492
CBU_0482
CS30
CS60
HS30
HS60

## Slide 4
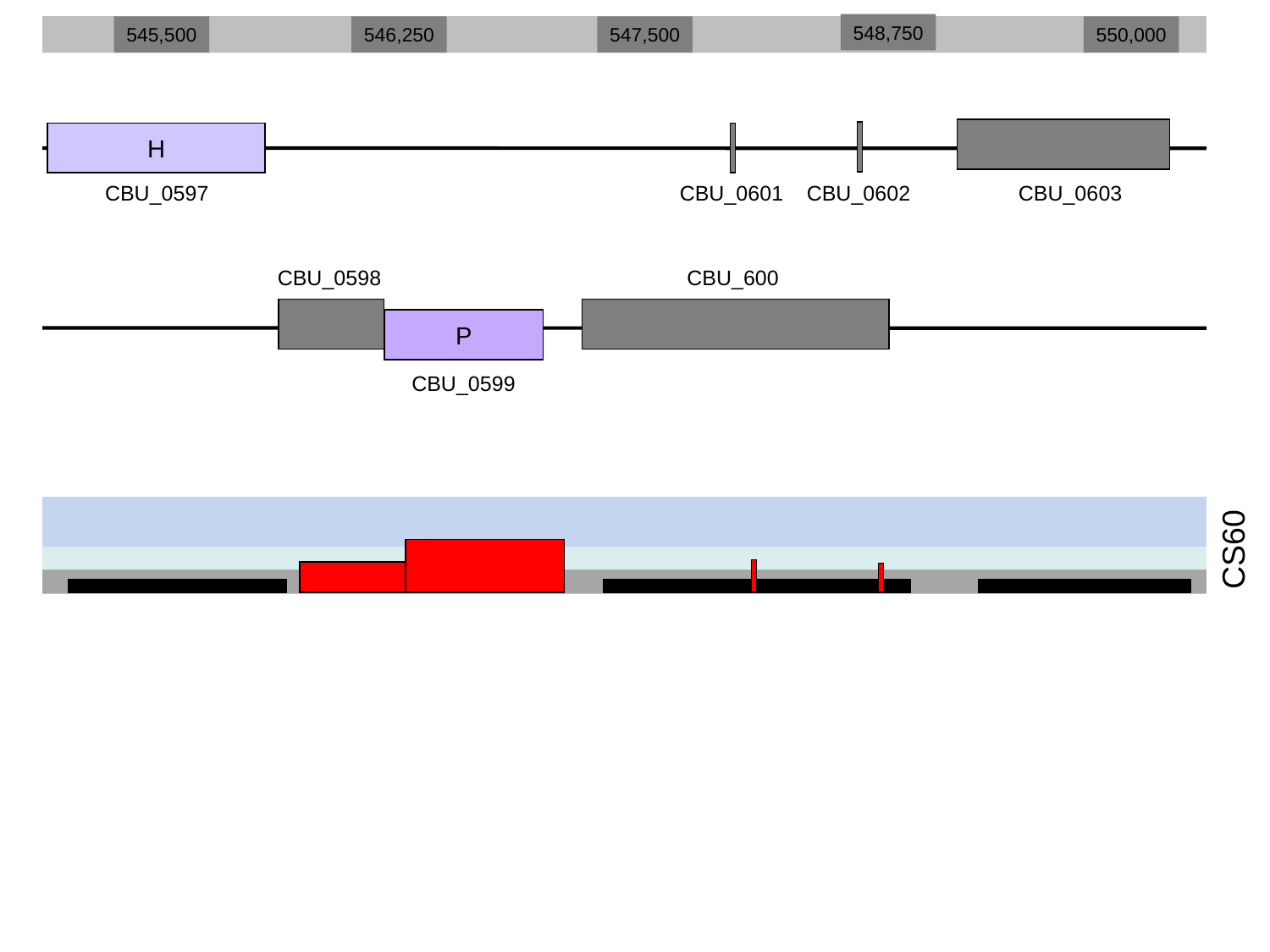

548,750
545,500
546,250
547,500
550,000
H
CBU_0597
CBU_0601
CBU_0602
CBU_0603
CBU_0598
CBU_600
P
CBU_0599
CS60

## Slide 5
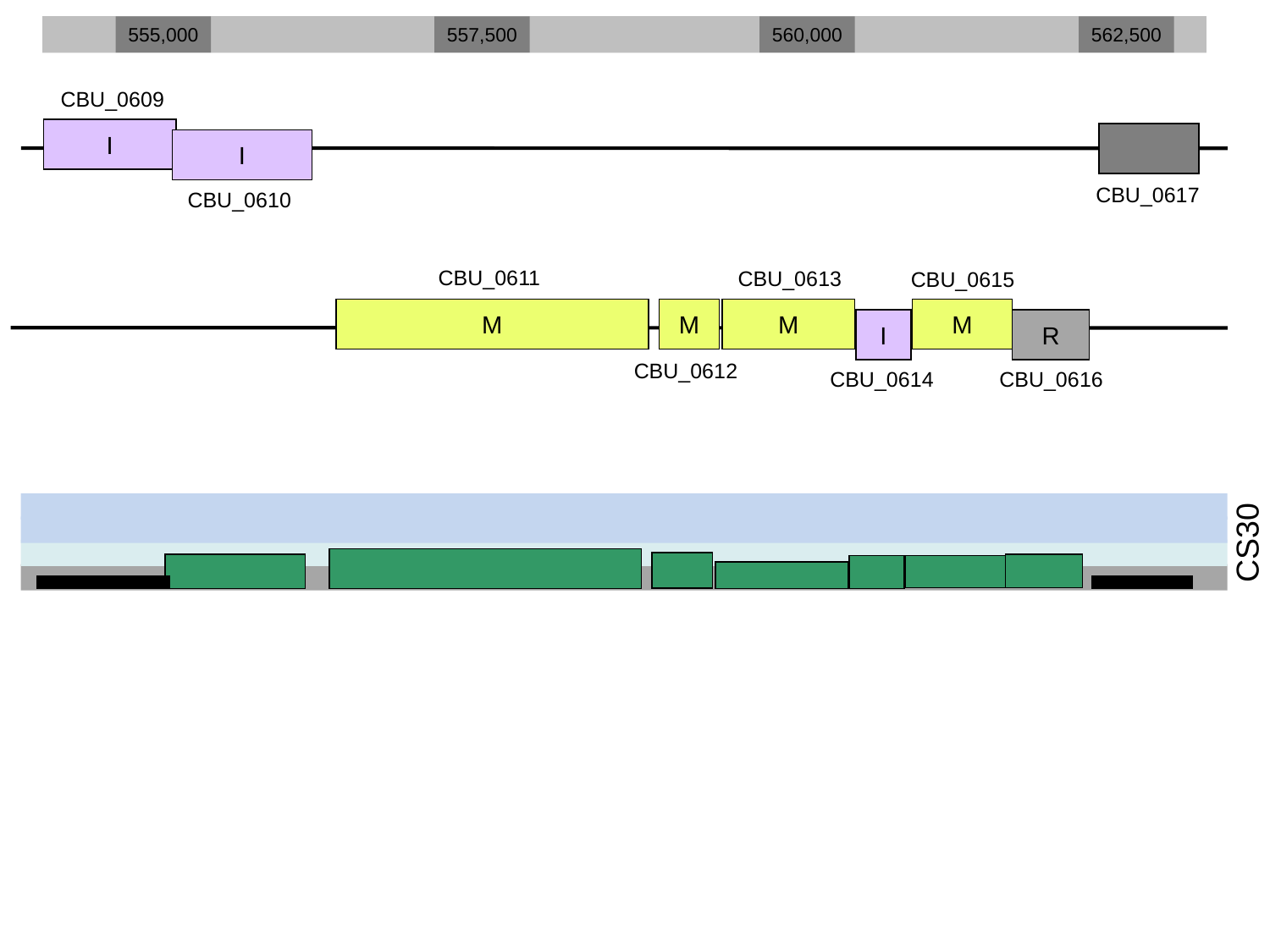

555,000
557,500
560,000
562,500
CBU_0609
I
I
CBU_0617
CBU_0610
CBU_0611
CBU_0613
CBU_0615
M
M
M
M
I
R
CBU_0612
CBU_0614
CBU_0616
CS30

## Slide 6
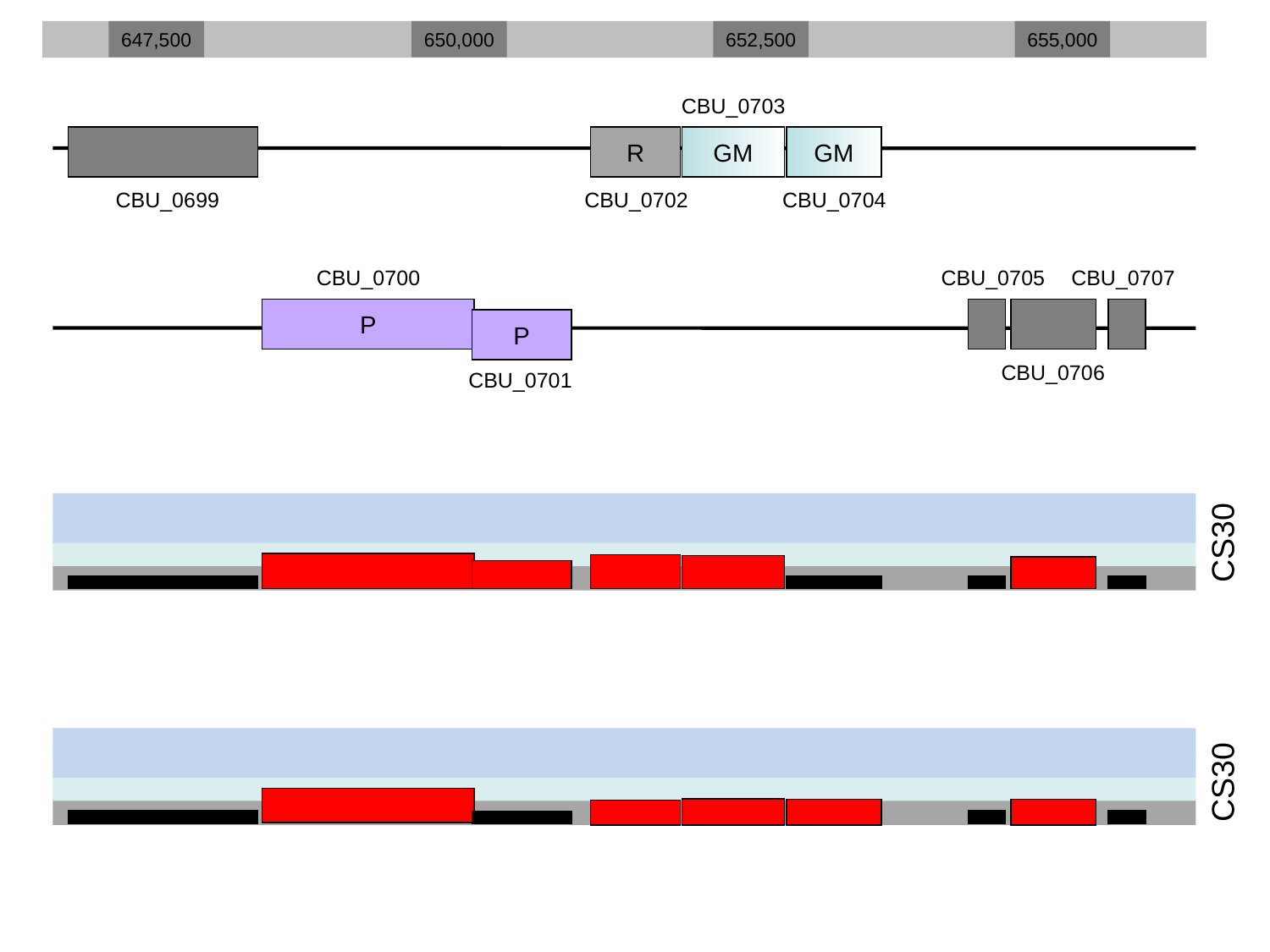

647,500
650,000
652,500
655,000
CBU_0703
R
GM
GM
CBU_0699
CBU_0702
CBU_0704
CBU_0700
CBU_0705
CBU_0707
P
P
CBU_0706
CBU_0701
CS30
CS30

## Slide 7
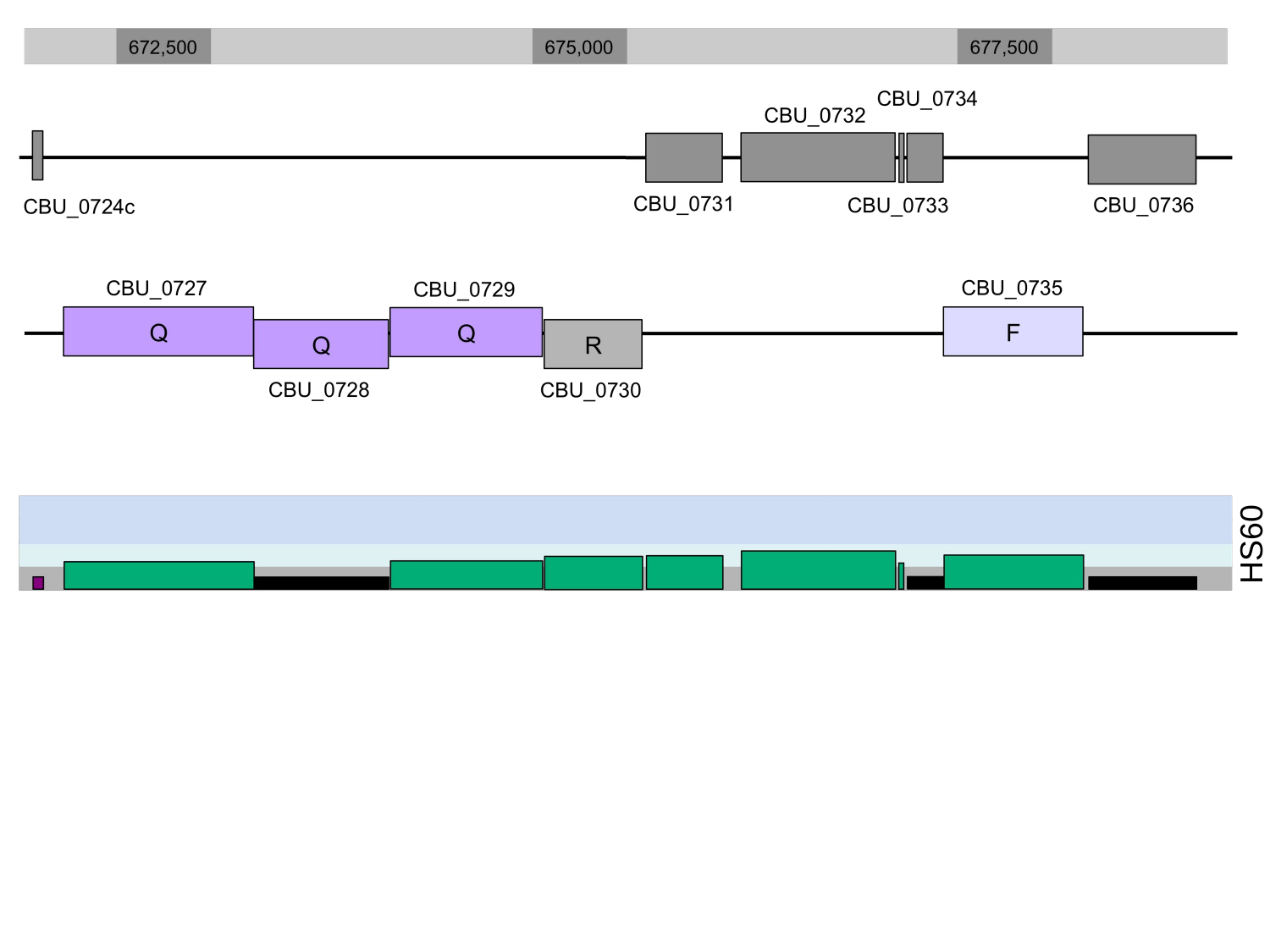

HS60

## Slide 8
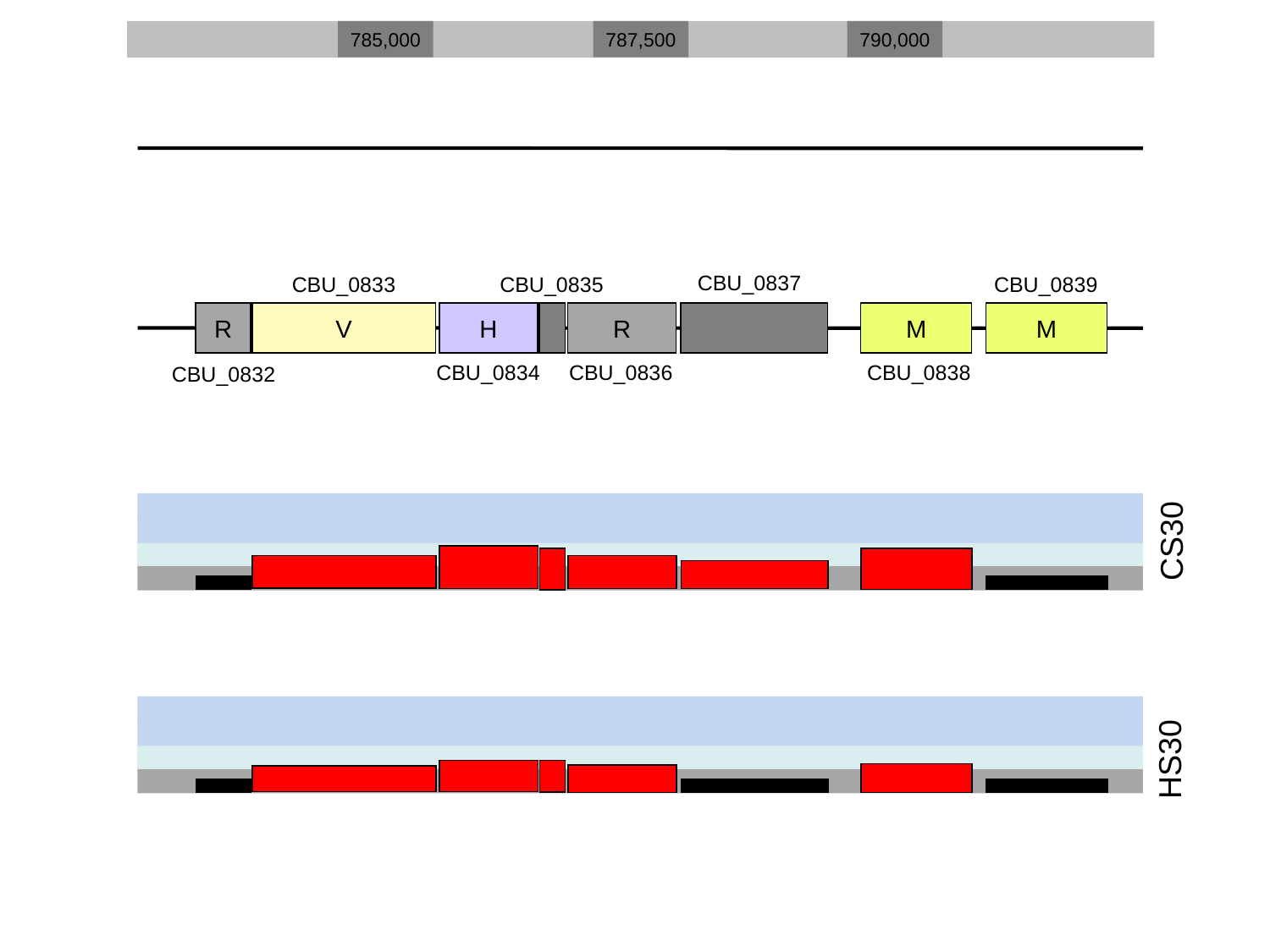

785,000
787,500
790,000
CBU_0837
CBU_0833
CBU_0835
CBU_0839
R
V
H
R
M
M
CBU_0834
CBU_0836
CBU_0838
CBU_0832
CS30
HS30

## Slide 9
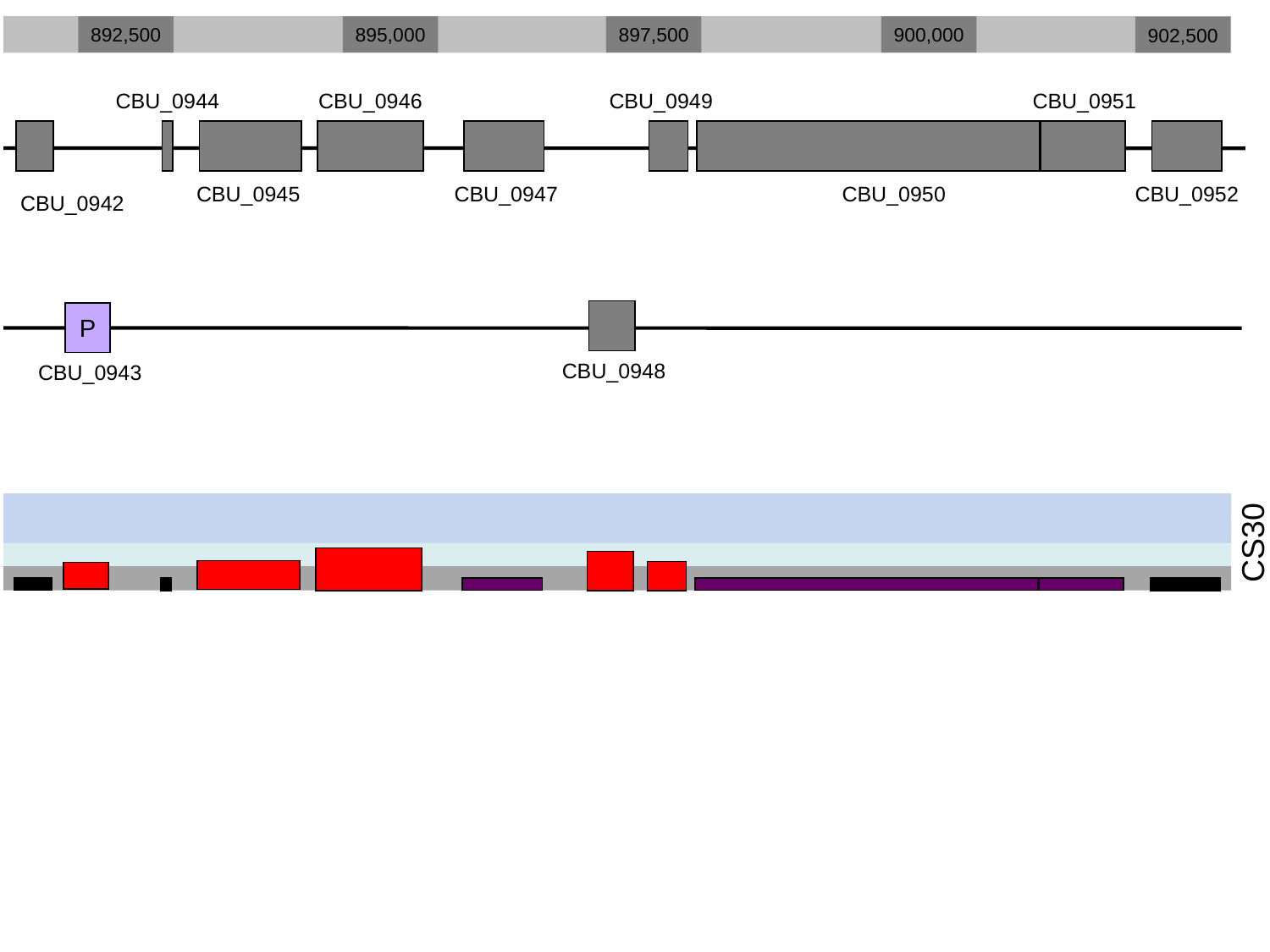

892,500
895,000
897,500
900,000
902,500
CBU_0944
CBU_0946
CBU_0949
CBU_0951
CBU_0945
CBU_0947
CBU_0950
CBU_0952
CBU_0942
P
CBU_0948
CBU_0943
CS30

## Slide 10
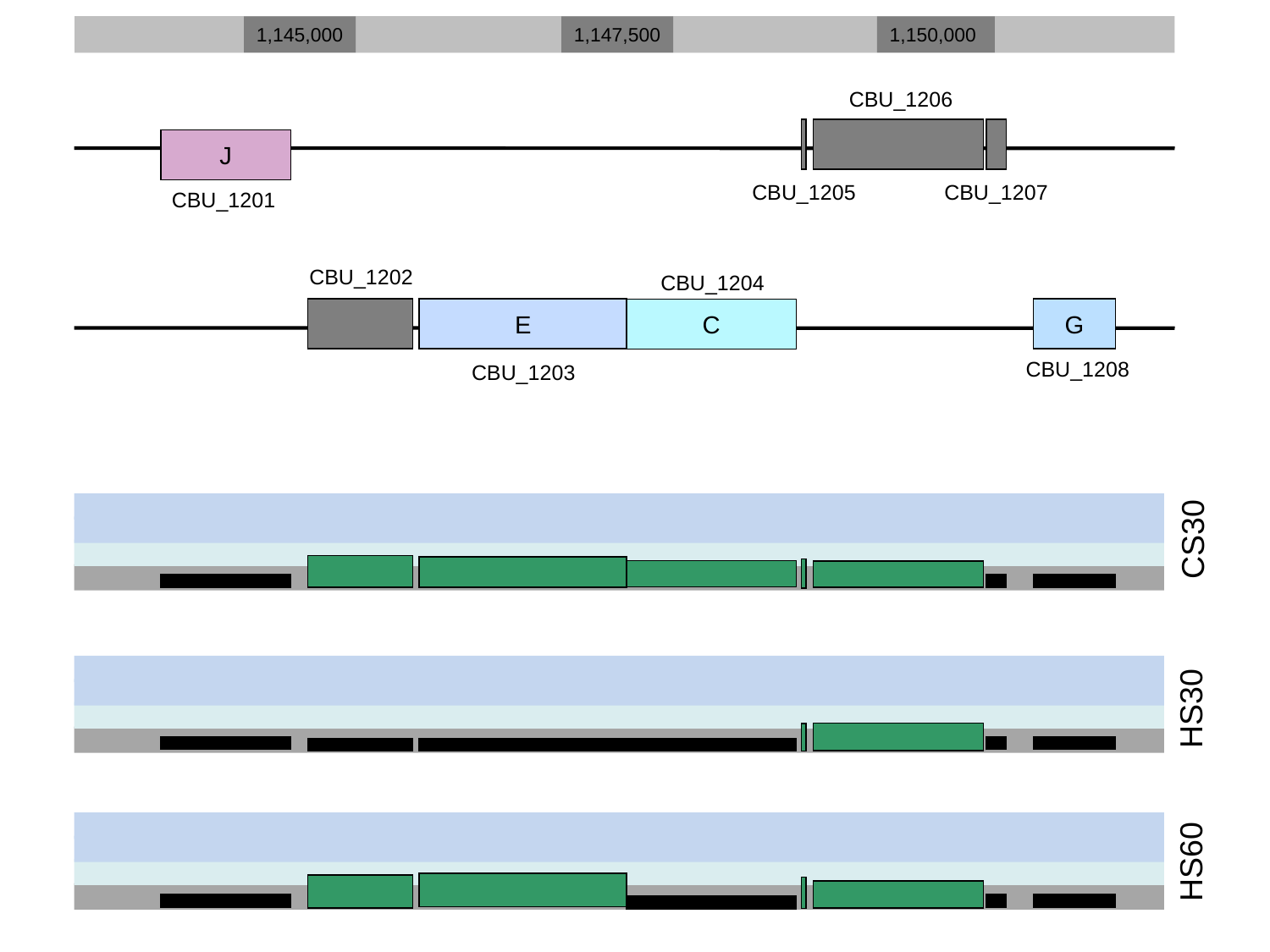

1,145,000
1,147,500
1,150,000
CBU_1206
J
CBU_1205
CBU_1207
CBU_1201
CBU_1202
CBU_1204
E
G
C
CBU_1208
CBU_1203
CS30
HS30
HS60

## Slide 11
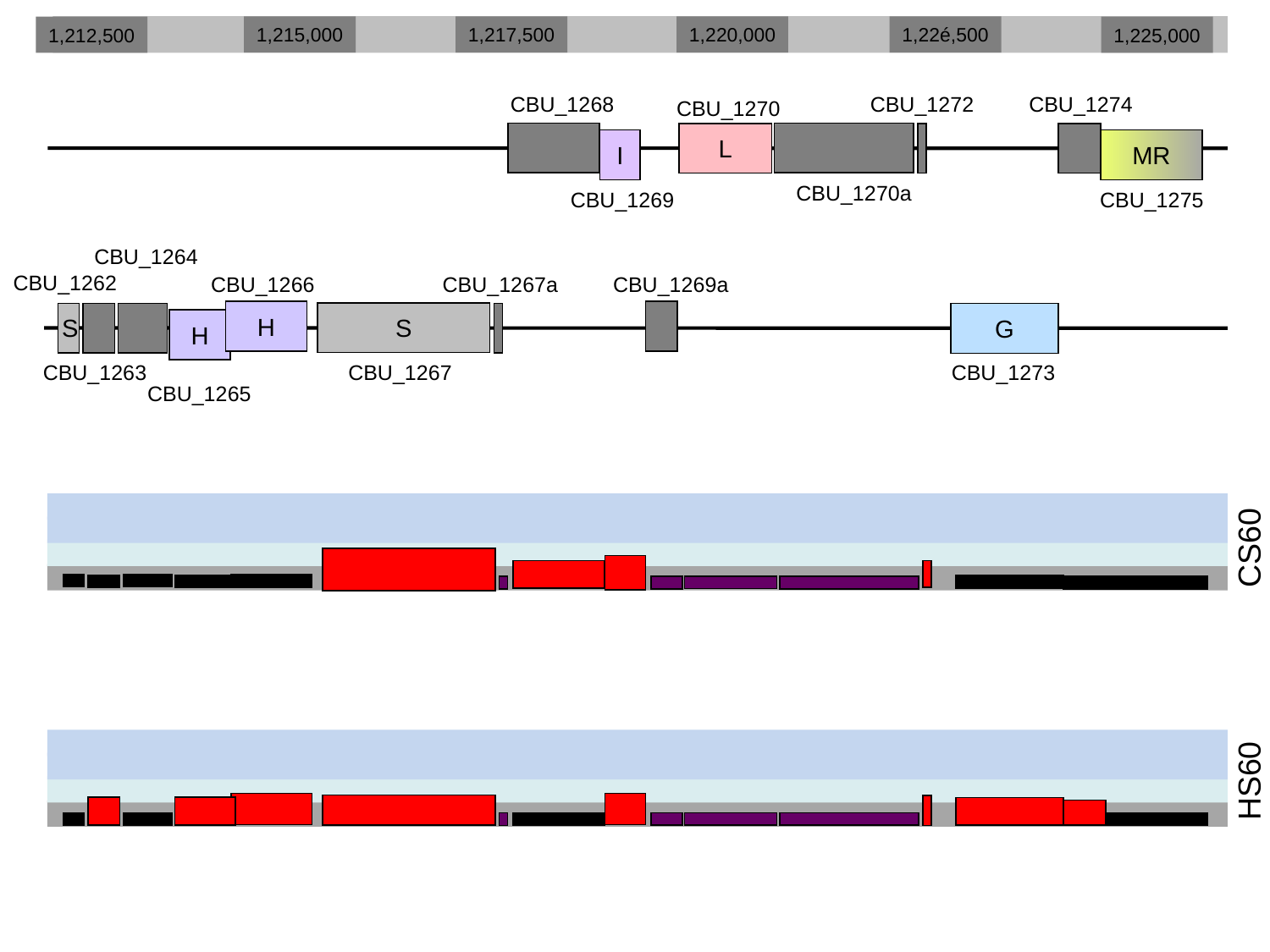

1,215,000
1,217,500
1,220,000
1,22é,500
1,212,500
1,225,000
CBU_1268
CBU_1272
CBU_1274
CBU_1270
L
I
MR
CBU_1270a
CBU_1269
CBU_1275
CBU_1264
CBU_1262
CBU_1266
CBU_1267a
CBU_1269a
H
S
G
S
H
CBU_1263
CBU_1267
CBU_1273
CBU_1265
CS60
HS60

## Slide 12
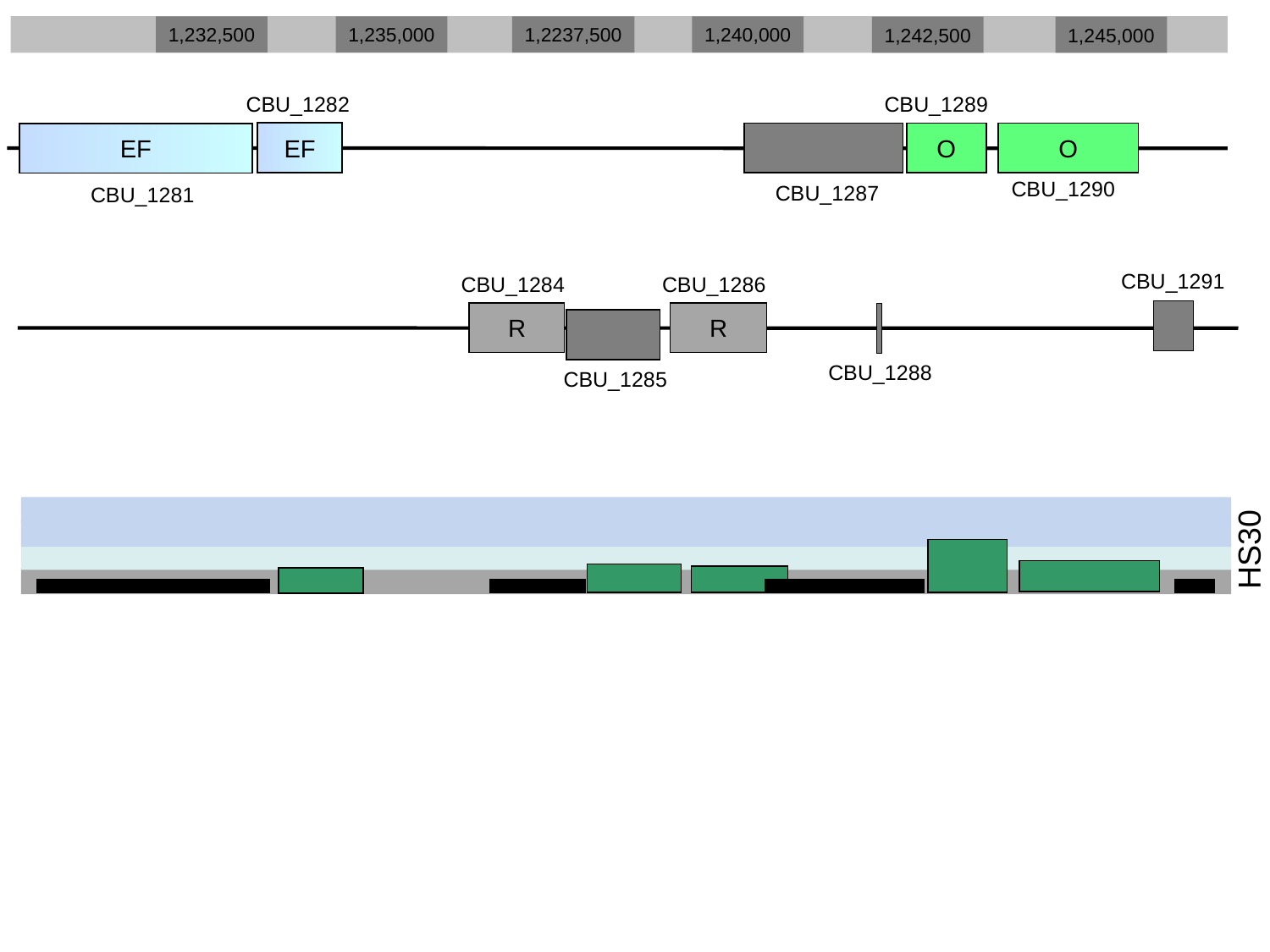

1,232,500
1,235,000
1,2237,500
1,240,000
1,242,500
1,245,000
CBU_1282
CBU_1289
EF
O
O
EF
CBU_1290
CBU_1287
CBU_1281
CBU_1291
CBU_1284
CBU_1286
R
R
CBU_1288
CBU_1285
HS30

## Slide 13
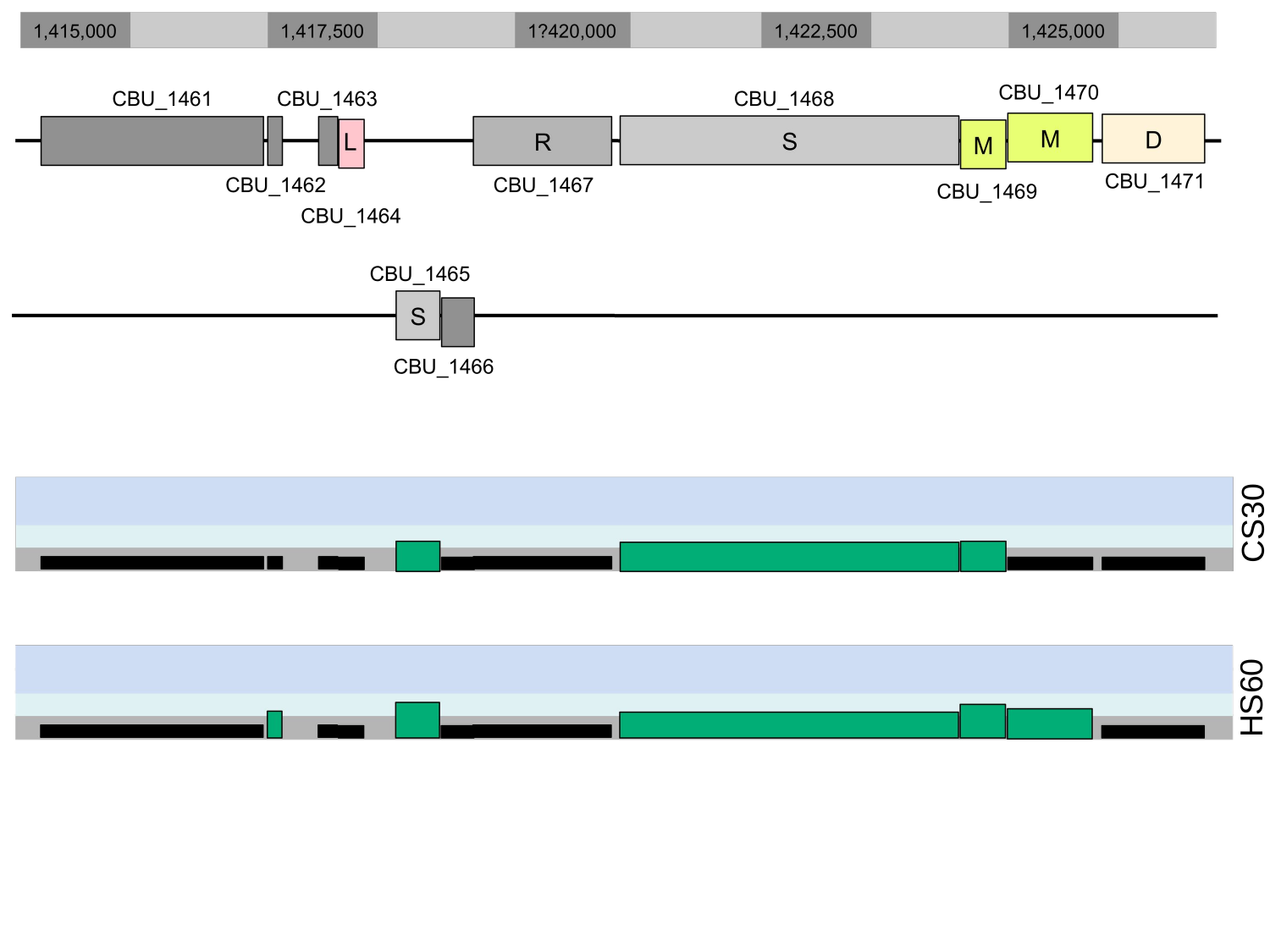

CS30
HS60

## Slide 14
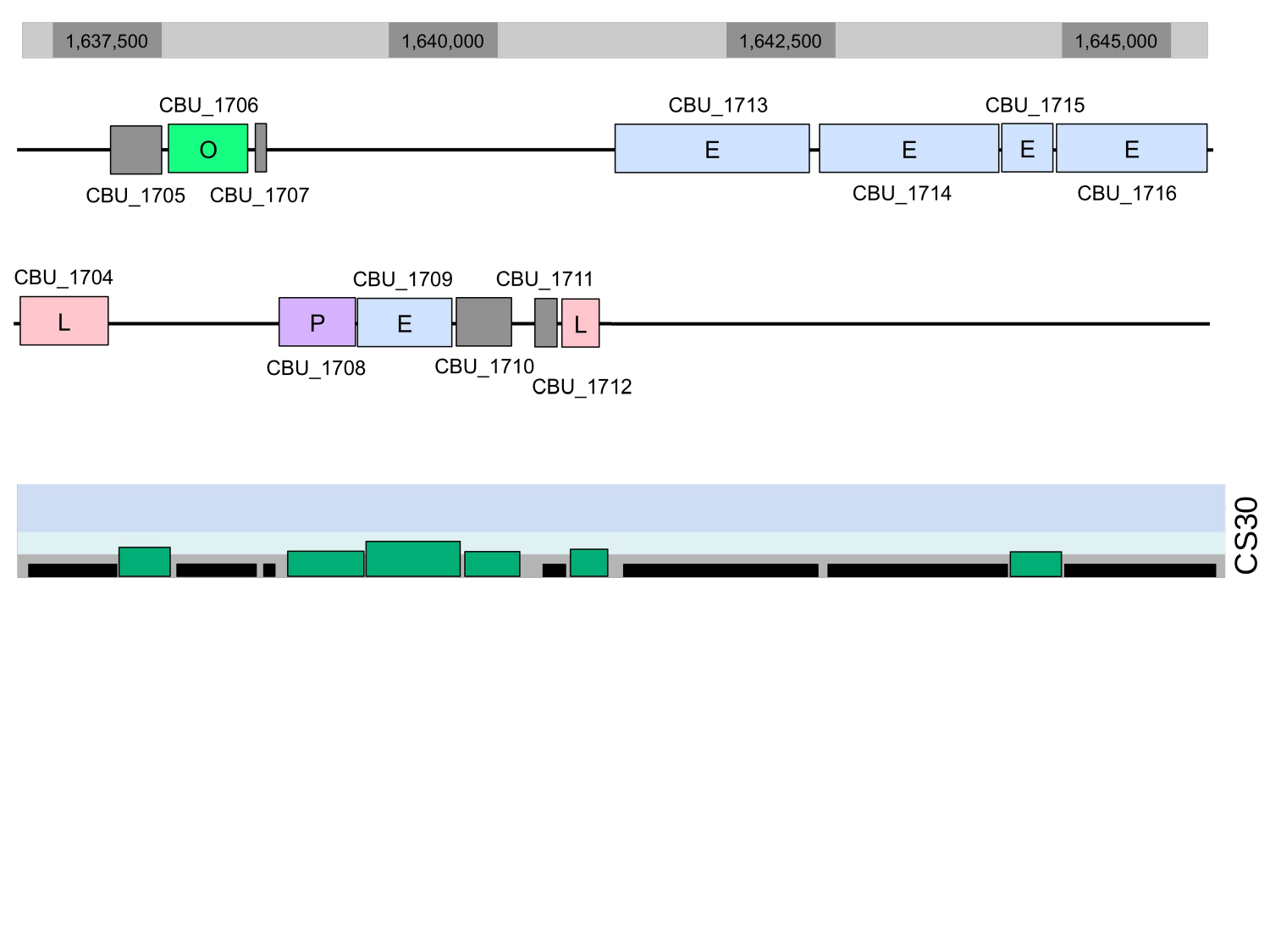

CS30

## Slide 15
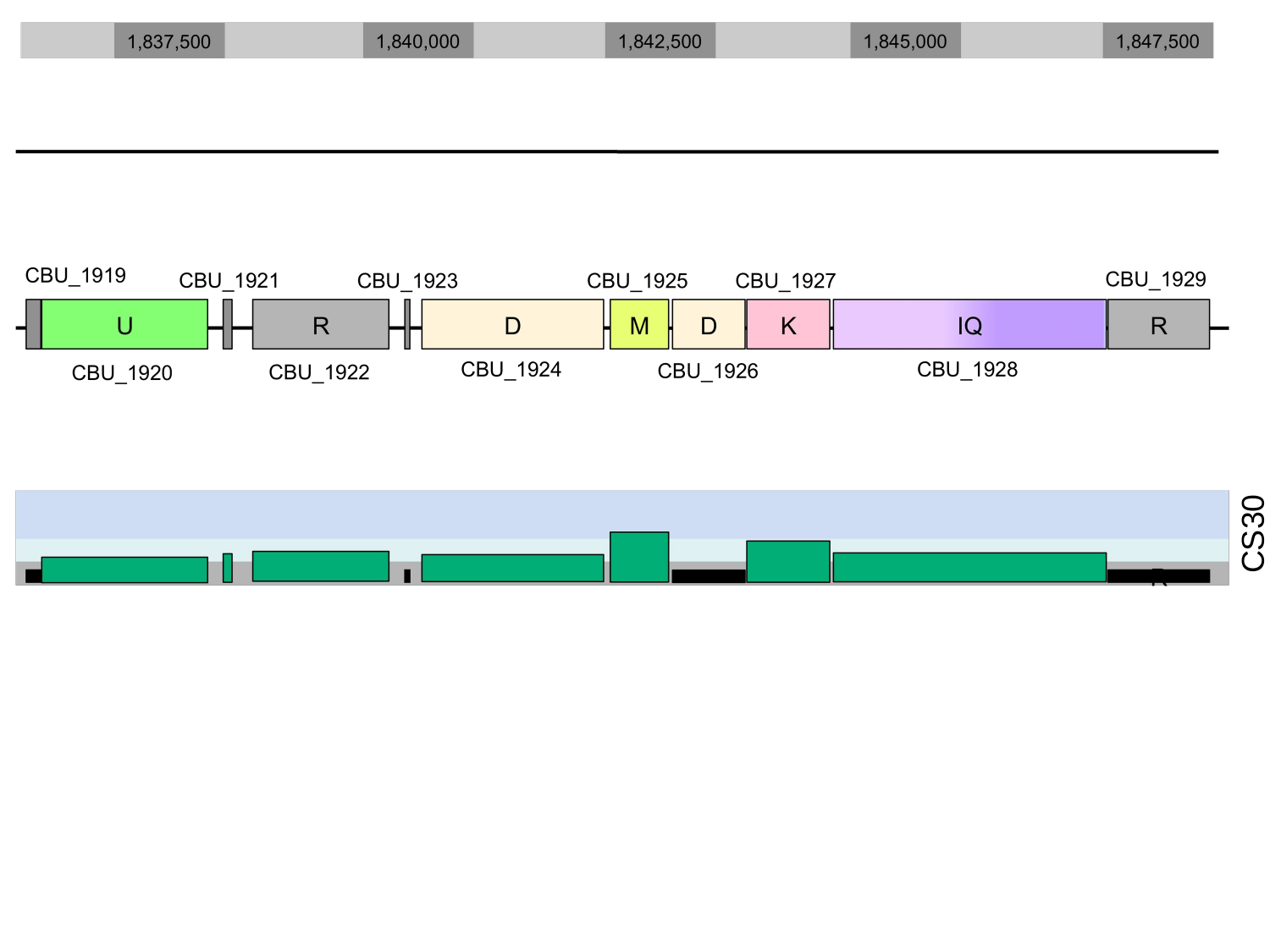

CS30

## Slide 16
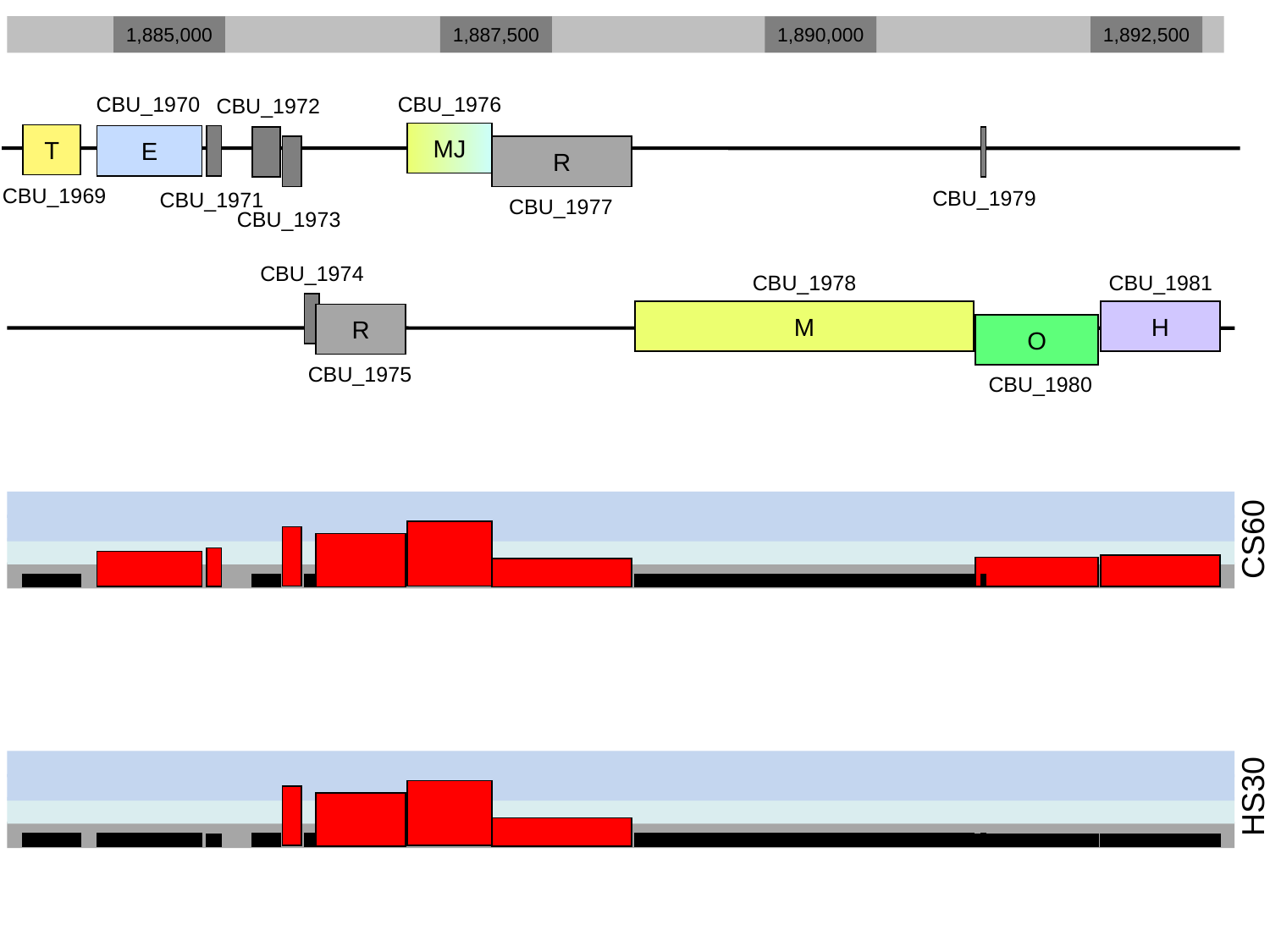

1,885,000
1,887,500
1,890,000
1,892,500
CBU_1970
CBU_1976
CBU_1972
MJ
T
E
R
CBU_1969
CBU_1979
CBU_1971
CBU_1977
CBU_1973
CBU_1974
CBU_1978
CBU_1981
M
H
R
O
CBU_1975
CBU_1980
CS60
HS30
