# Supplementary material for: Coxiella burnetii Transcriptional Analysis Reveals Serendipity Clusters of Regulation in Intracellular Bacteria
Source: PLoS One. 2010 Dec 21;5(12):e15321. doi: 10.1371/journal.pone.0015321 (PMC3006202; doi:10.1371/journal.pone.0015321)
Supplement: Figure S3 — Coxiella burnetii gene network connections. (PPT) [file pone.0015321.s003.ppt]

## Slide 1
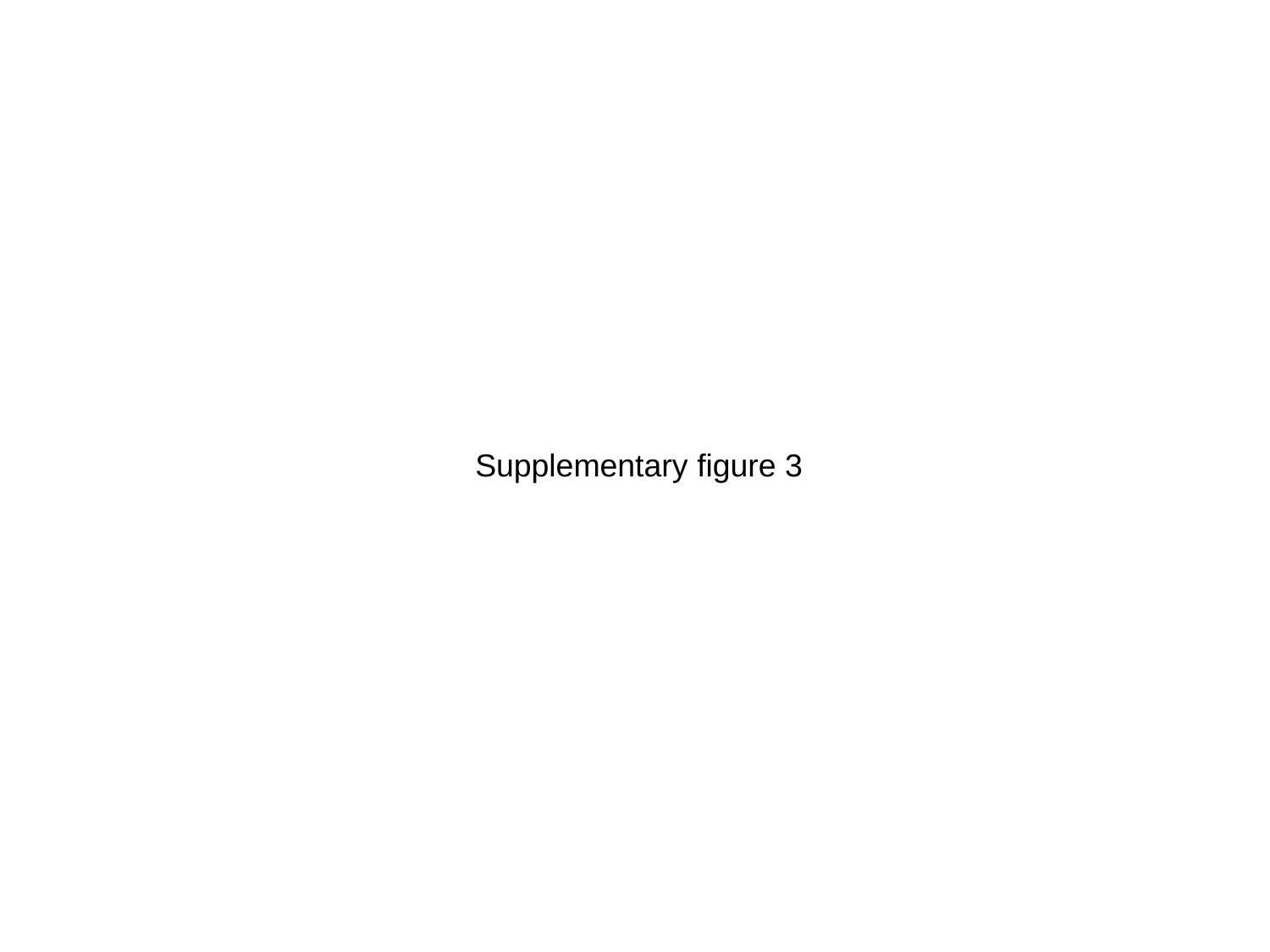

Supplementary figure 3

## Slide 2
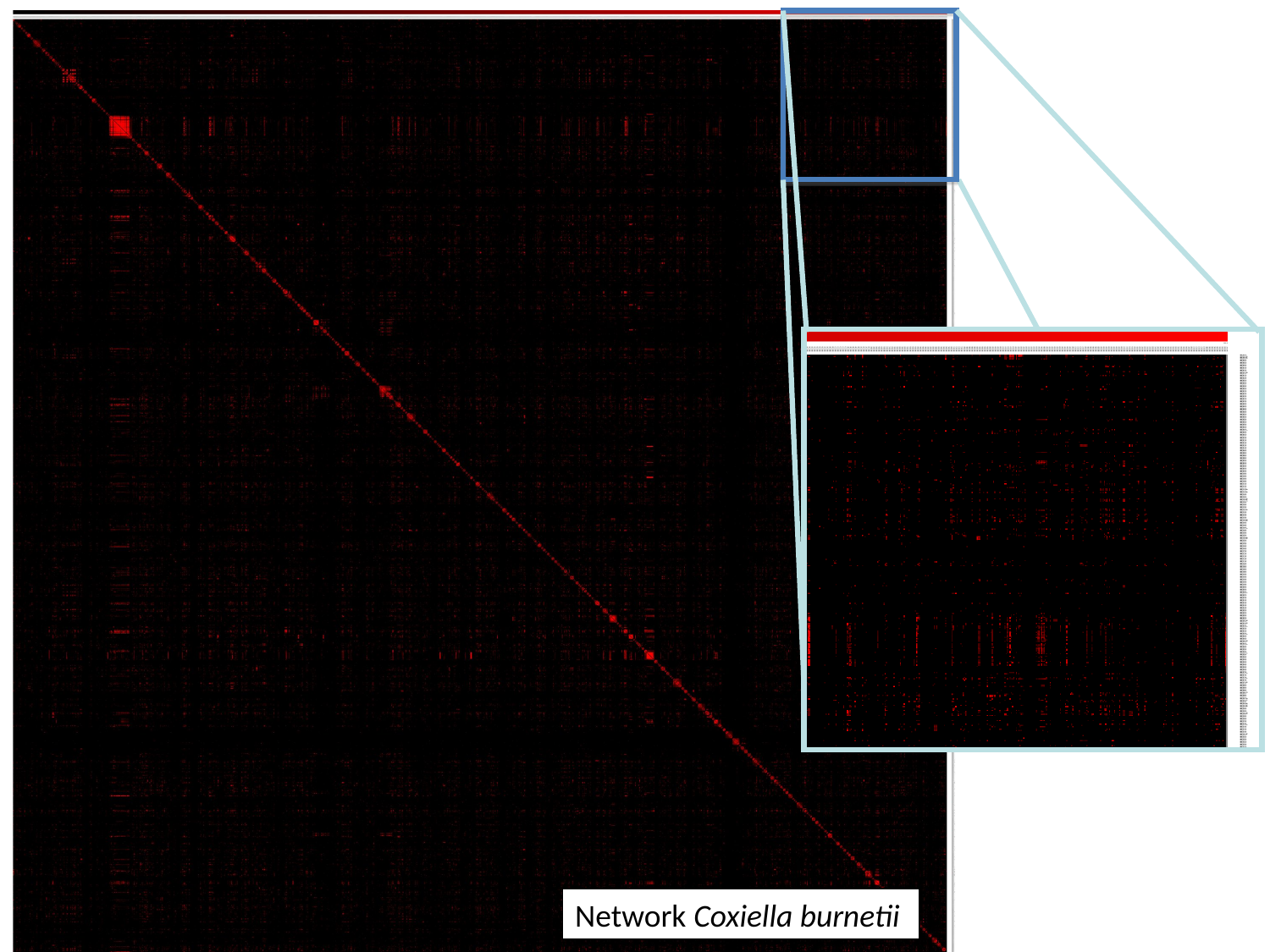

#
Network Coxiella burnetii

## Slide 3
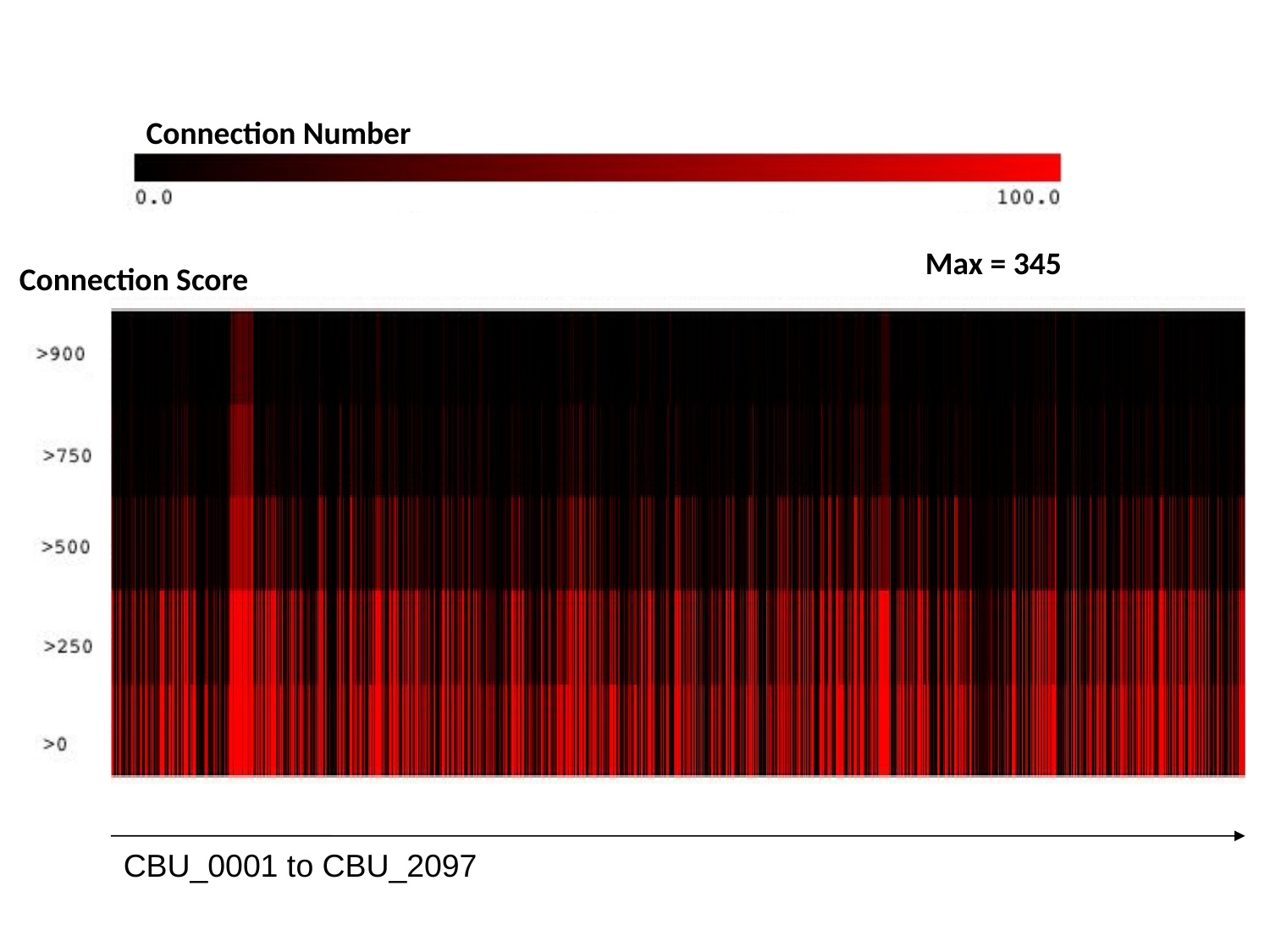

Connection Number
Max = 345
Connection Score
CBU_0001 to CBU_2097
