# Supplementary material for: Coxiella burnetii Transcriptional Analysis Reveals Serendipity Clusters of Regulation in Intracellular Bacteria
Source: PLoS One. 2010 Dec 21;5(12):e15321. doi: 10.1371/journal.pone.0015321 (PMC3006202; doi:10.1371/journal.pone.0015321)
Supplement: Figure S4 — Differentially expressed gene networks. (PPT) [file pone.0015321.s004.ppt]

## Slide 1
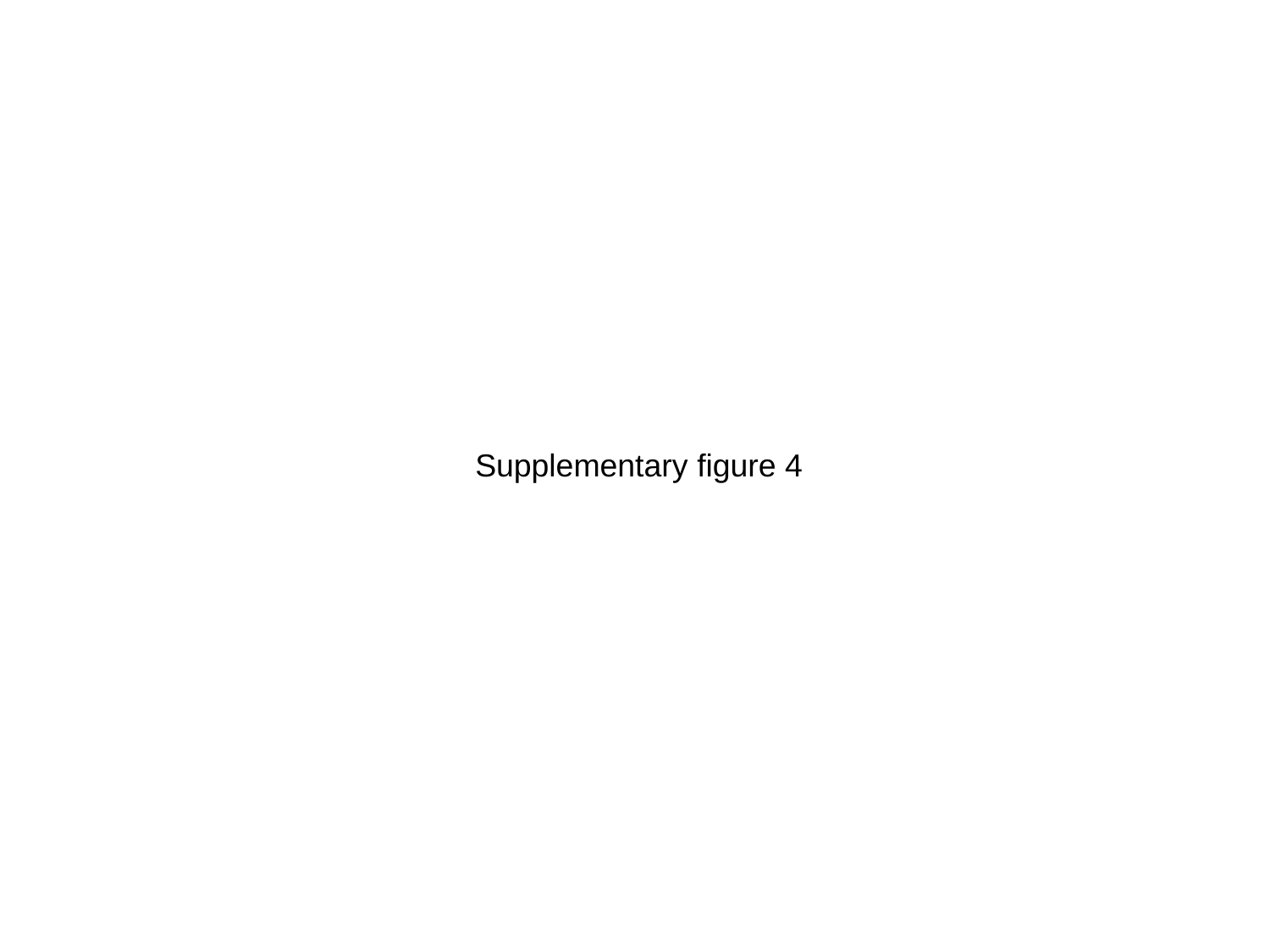

Supplementary figure 4

## Slide 2
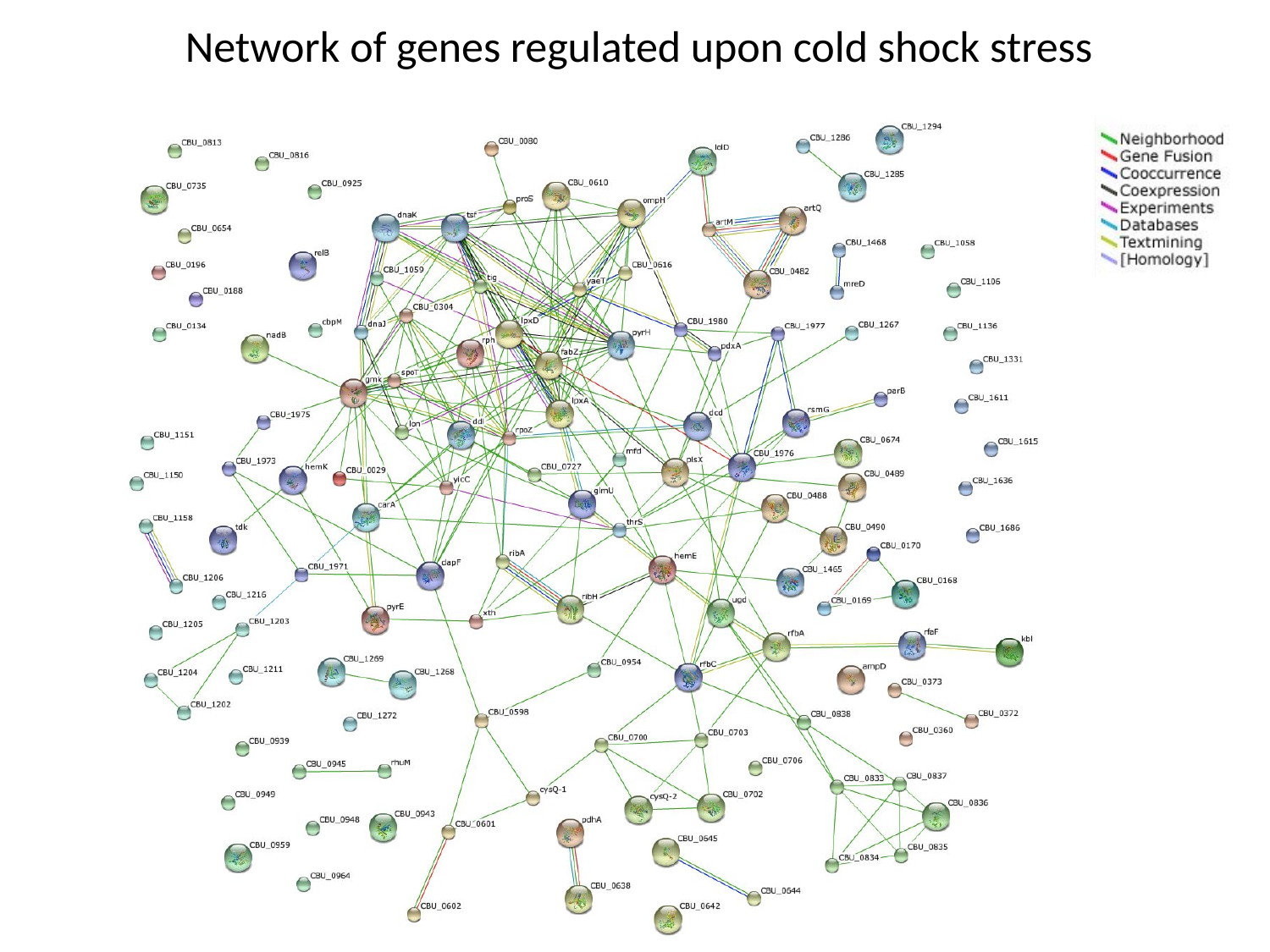

Network of genes regulated upon cold shock stress

## Slide 3
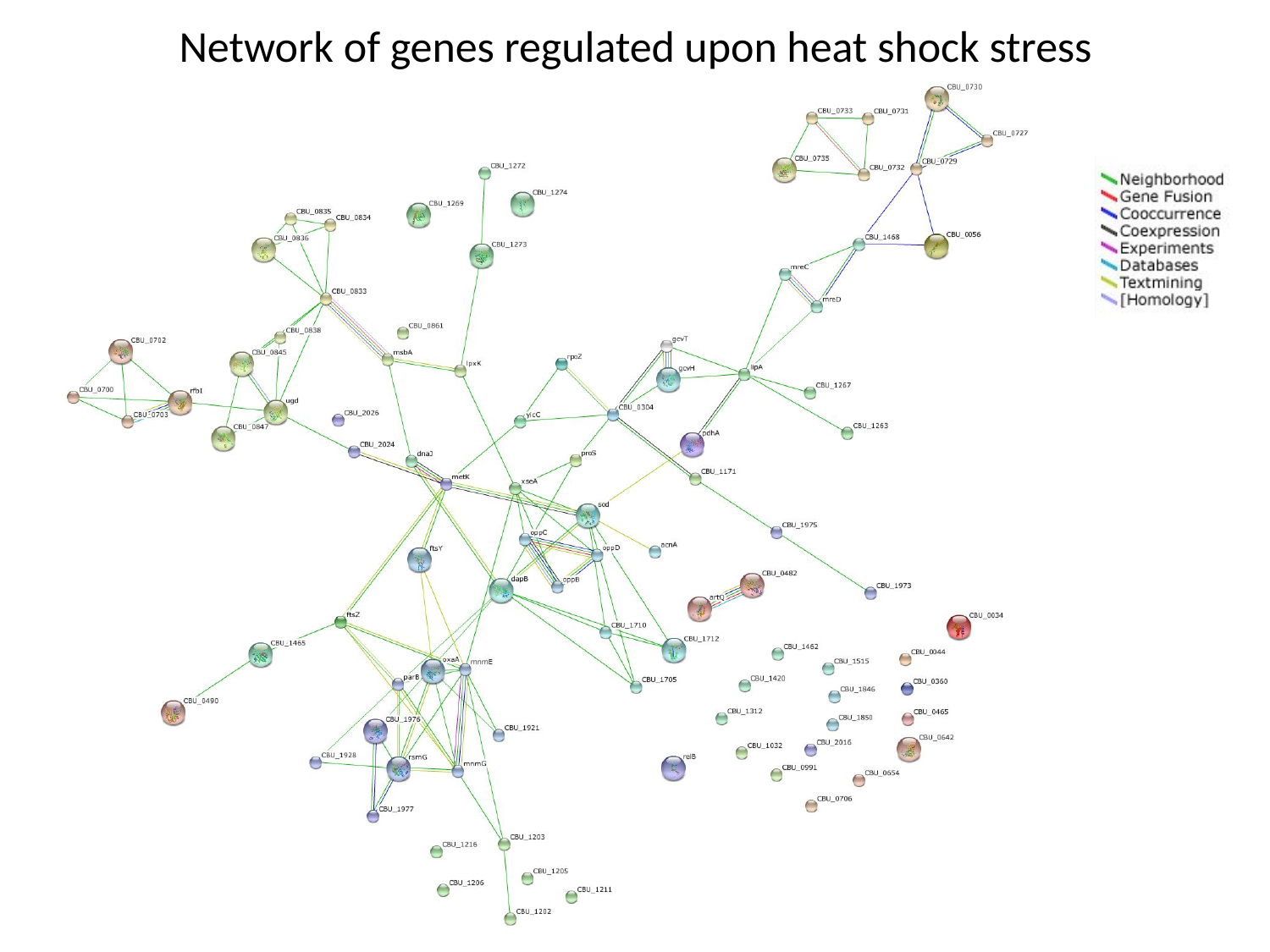

Network of genes regulated upon heat shock stress
