# Supplementary material for: Coxiella burnetii Transcriptional Analysis Reveals Serendipity Clusters of Regulation in Intracellular Bacteria
Source: PLoS One. 2010 Dec 21;5(12):e15321. doi: 10.1371/journal.pone.0015321 (PMC3006202; doi:10.1371/journal.pone.0015321)
Supplement: Figure S5 — Promoter sequence analysis. (PPT) [file pone.0015321.s005.ppt]

## Slide 1
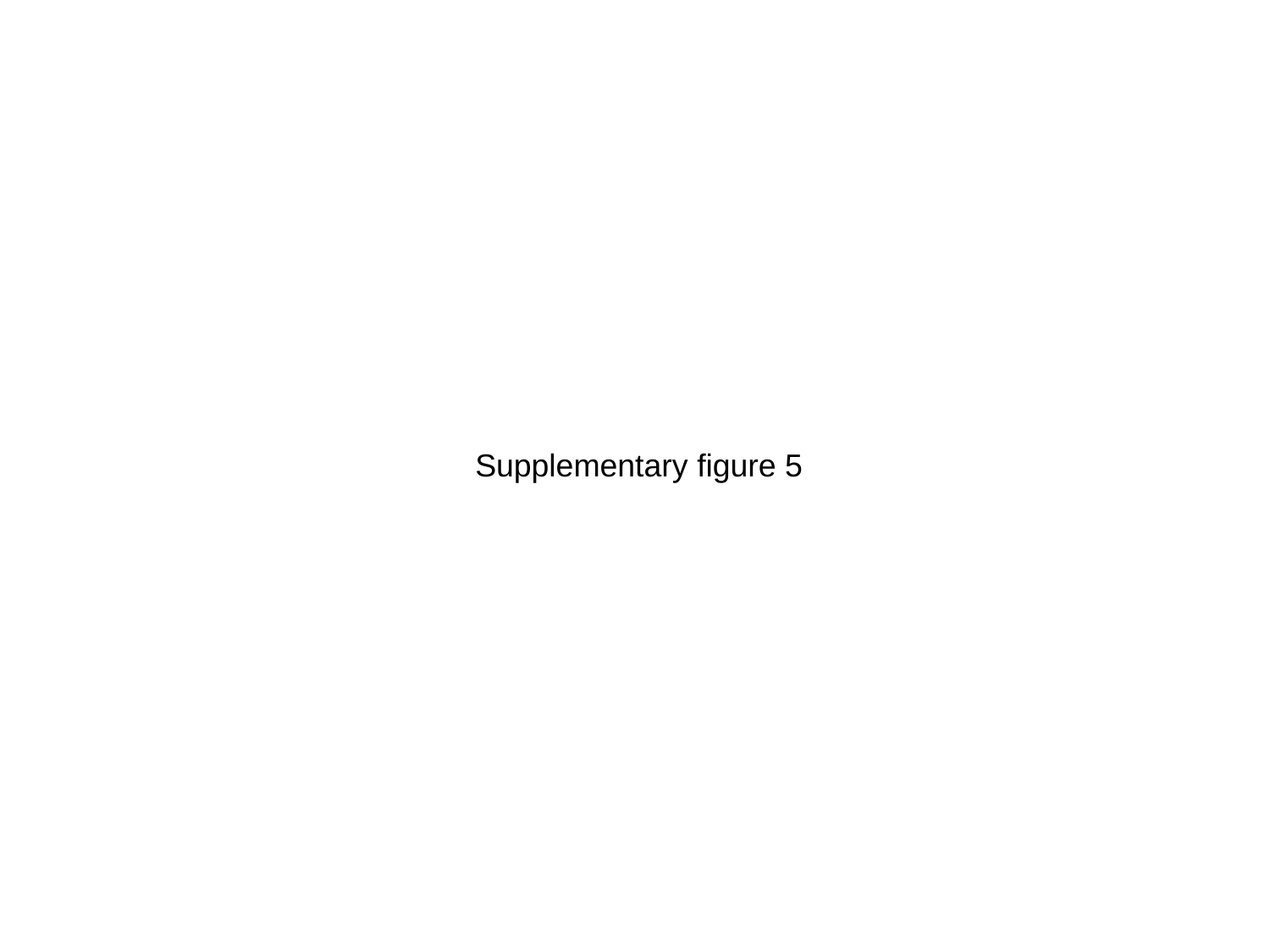

Supplementary figure 5

## Slide 2
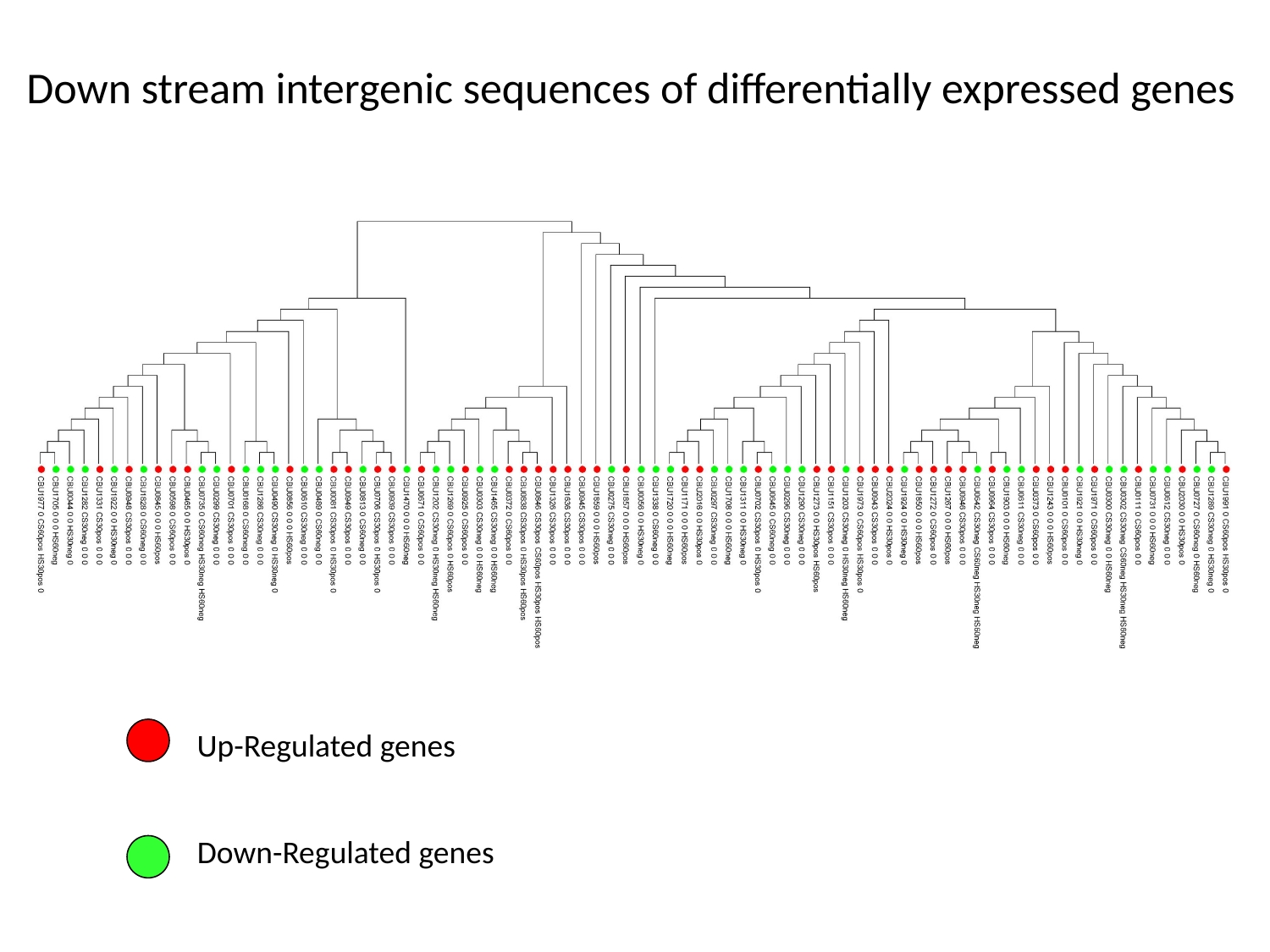

Down stream intergenic sequences of differentially expressed genes
#
Up-Regulated genes
Down-Regulated genes

## Slide 3
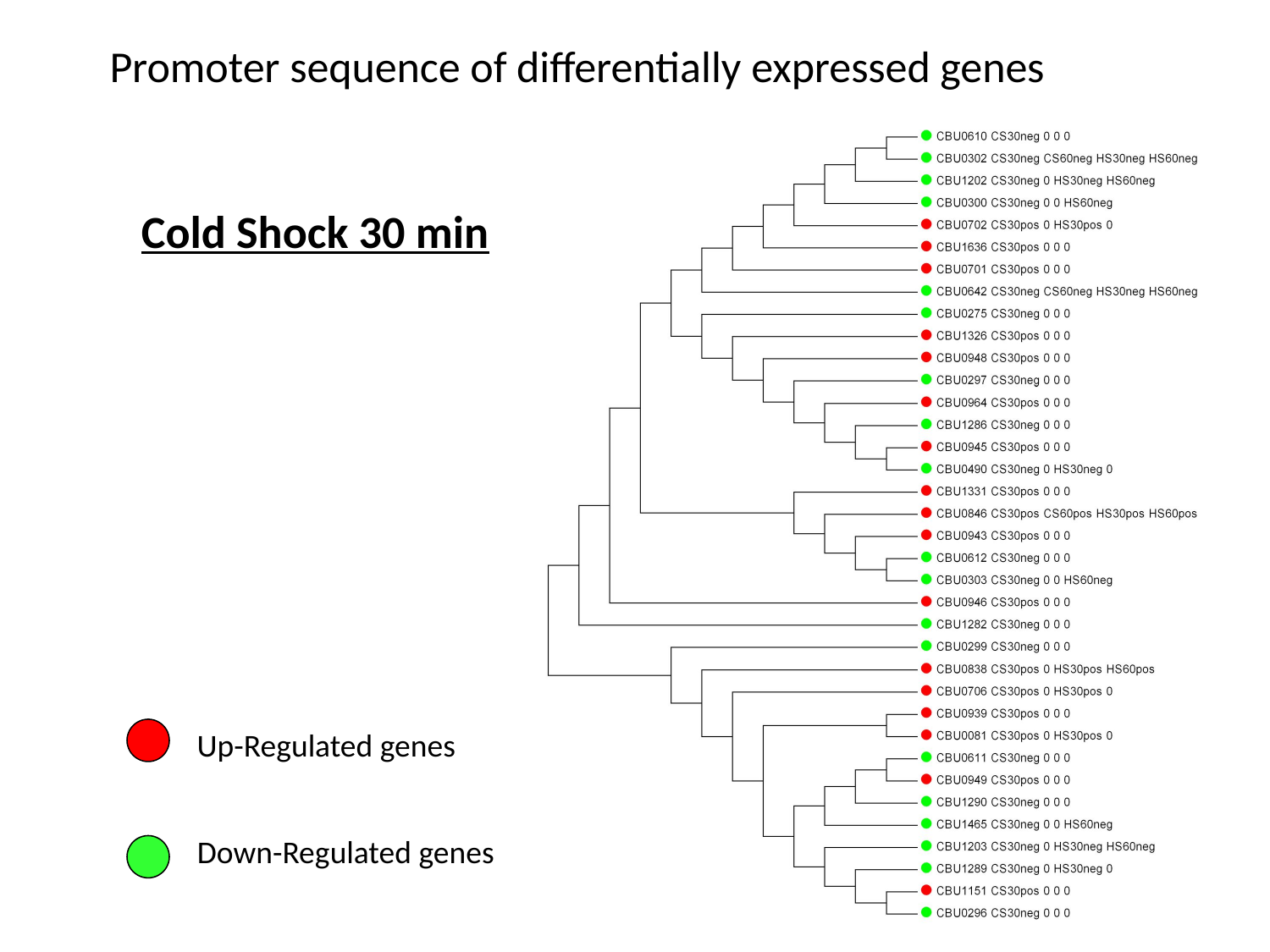

Promoter sequence of differentially expressed genes
Cold Shock 30 min
Up-Regulated genes
Down-Regulated genes

## Slide 4
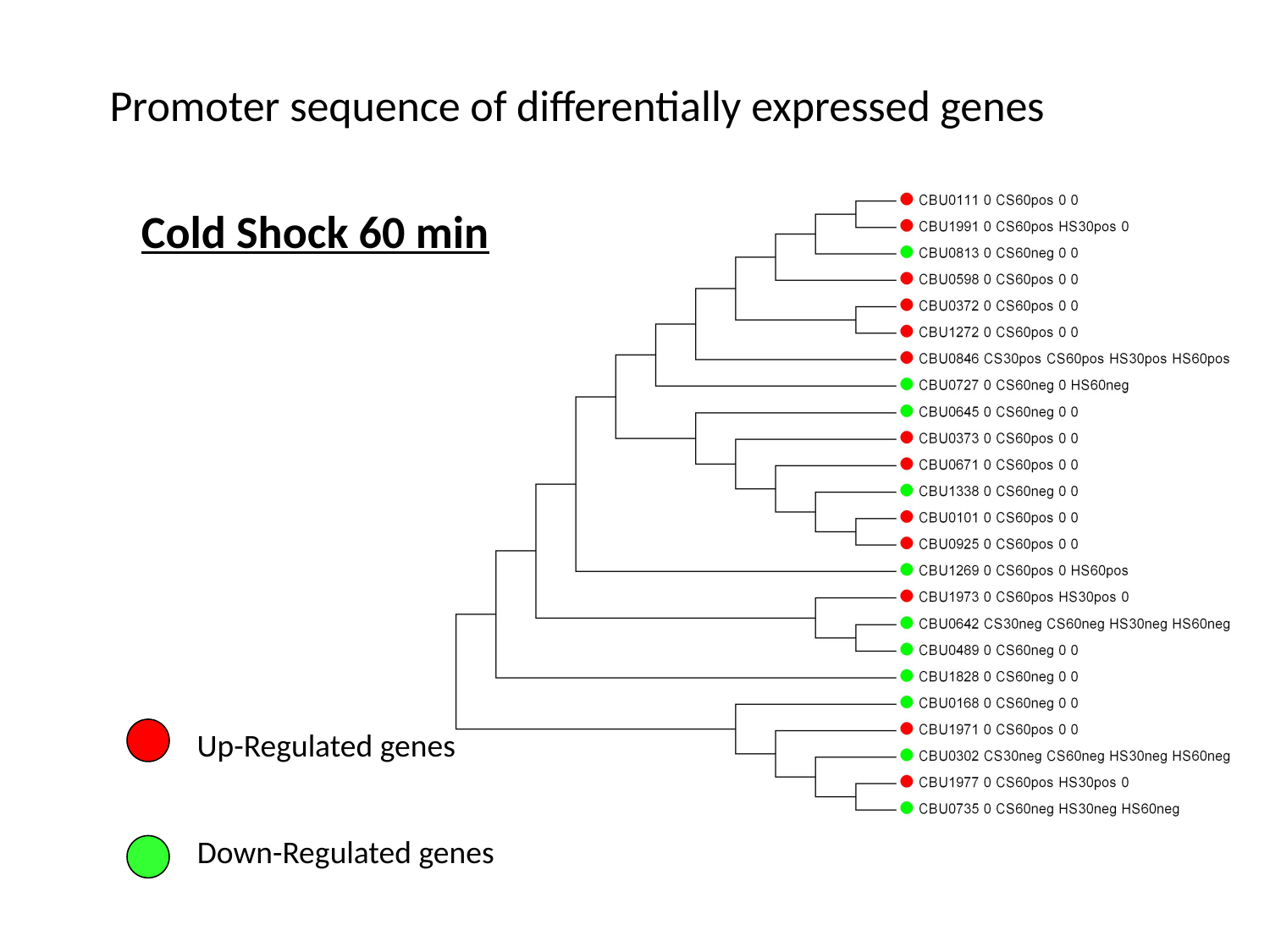

Promoter sequence of differentially expressed genes
Cold Shock 60 min
#
Up-Regulated genes
Down-Regulated genes

## Slide 5
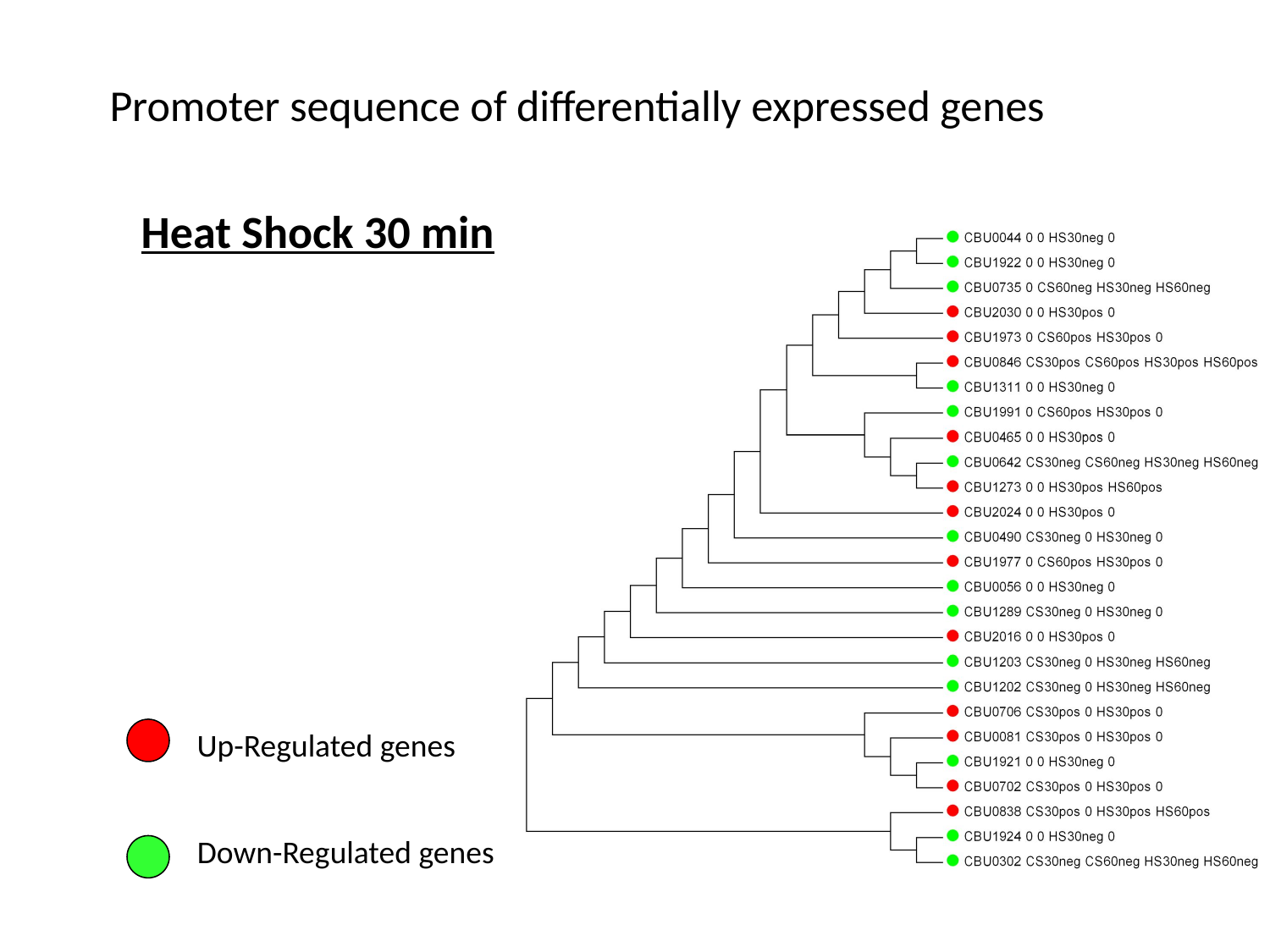

Promoter sequence of differentially expressed genes
Heat Shock 30 min
Up-Regulated genes
Down-Regulated genes

## Slide 6
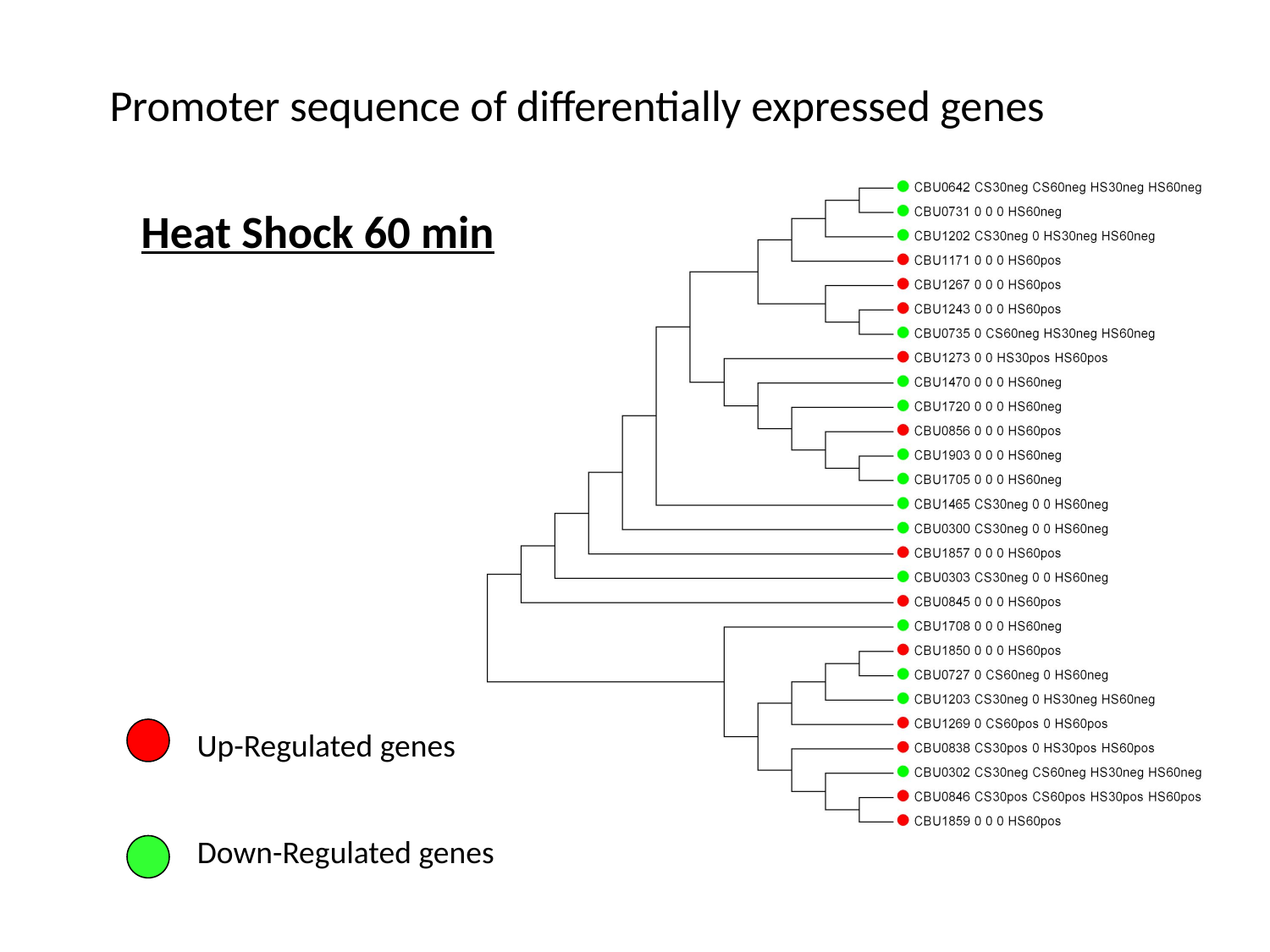

Promoter sequence of differentially expressed genes
Heat Shock 60 min
Up-Regulated genes
Down-Regulated genes

## Slide 7
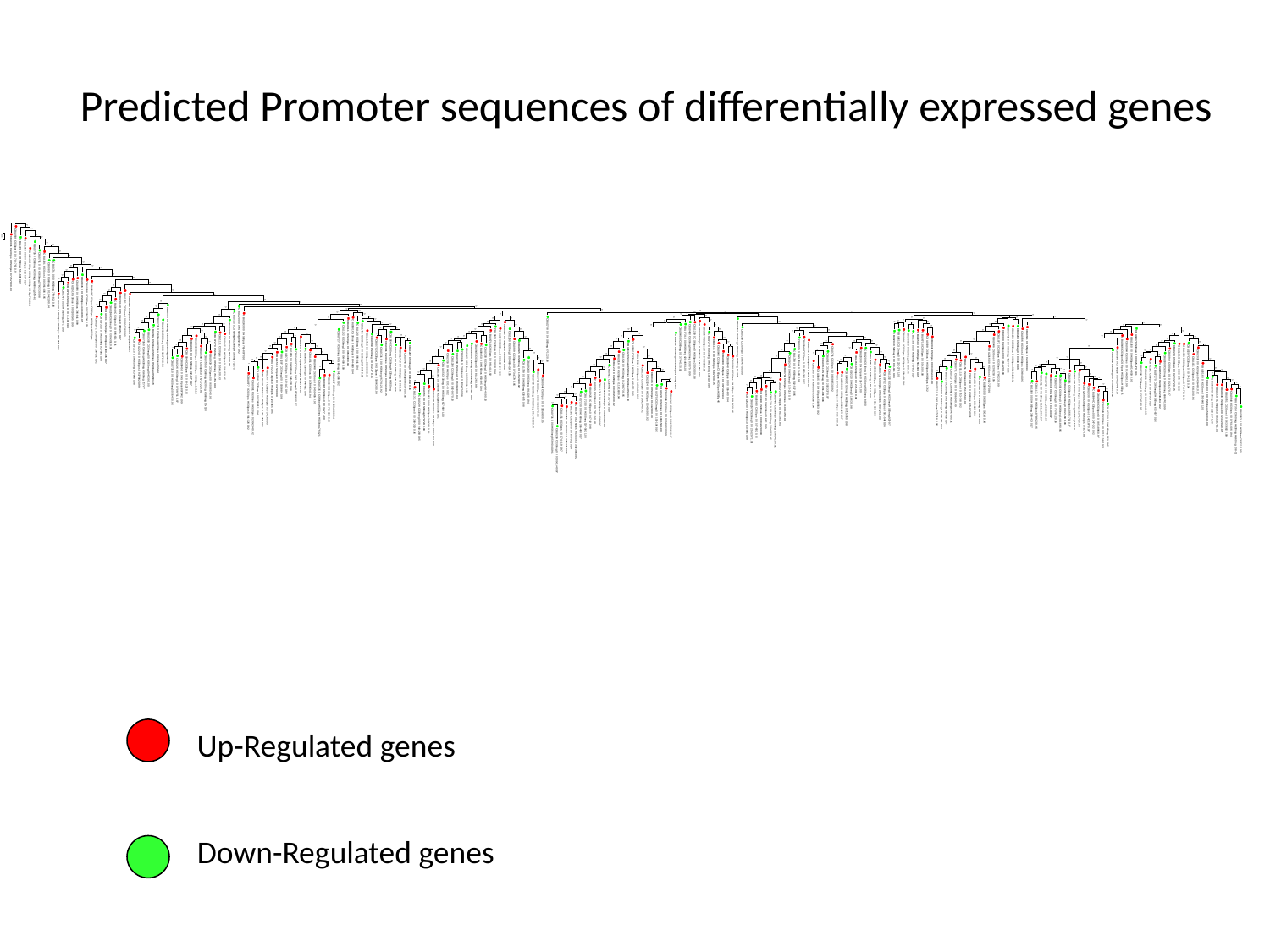

Predicted Promoter sequences of differentially expressed genes
Up-Regulated genes
Down-Regulated genes
